# s-HBEGF/SIRT1 circuit-dictated crosstalk between vascular endothelial cells and keratinocytes mediates sorafenib-induced hand–foot skin reaction that can be reversed by nicotinamide

**DOI:** 10.1038/s41422-020-0309-6

**Published:** 2020-04-15

**Authors:** Peihua Luo, Hao Yan, Xueqin Chen, Ying Zhang, Ziying Zhao, Ji Cao, Yi Zhu, Jiangxia Du, Zhifei Xu, Xiaochen Zhang, Su Zeng, Bo Yang, Shenglin Ma, Qiaojun He

**Affiliations:** 1 Institute of Pharmacology & Toxicology, College of Pharmaceutical Sciences, Zhejiang University, Hangzhou, 310058 Zhejiang China; 2 Department of Oncology, Key Laboratory of Clinical Cancer Pharmacology and Toxicology Research of Zhejiang Province, Affiliated Hangzhou First People’s Hospital, Zhejiang University School of Medicine, Hangzhou, 310006 Zhejiang China; 3 Department of Oncology, The First Affiliated Hospital, School of Medicine, Zhejiang University, Hangzhou, 310000 Zhejiang China; 4 Institute of Drug Metabolism and Pharmaceutical Analysis, College of Pharmaceutical Sciences, Zhejiang University, Hangzhou, 310058 Zhejiang China

**Keywords:** Mechanisms of disease, Cancer therapy

## Abstract

Hand–foot skin reaction (HFSR), among the most significant adverse effects of sorafenib, has been limiting the clinical benefits of this frontline drug in treating various malignant tumors. The mechanism underlying such toxicity remains poorly understood, hence the absence of effective intervention strategies. In the present study, we show that vascular endothelial cells are the primary cellular target of sorafenib-induced HFSR wherein soluble heparin-binding epidermal growth factor (s-HBEGF) mediates the crosstalk between vascular endothelial cells and keratinocytes. Mechanistically, s-HBEGF released from vascular endothelial cells activates the epidermal growth factor receptor (EGFR) on keratinocytes and promotes the phosphorylation of c-Jun N-terminal kinase 2 (JNK2), which stabilizes sirtuin 1 (SIRT1), an essential keratinization inducer, and ultimately gives rise to HFSR. The administration of s-HBEGF in vivo could sufficiently induce hyper-keratinization without sorafenib treatment. Furthermore, we report that HBEGF neutralization antibody, *Sirt1* knockdown, and a classic SIRT1 inhibitor nicotinamide could all significantly reduce the sorafenib-induced HFSR in the mouse model. It is noteworthy that nicotinic acid, a prodrug of nicotinamide, could substantially reverse the sorafenib-induced HFSR in ten patients in a preliminary clinical study. Collectively, our findings reveal the mechanism of vascular endothelial cell-promoted keratinization in keratinocytes and provide a potentially promising therapeutic strategy for the treatment of sorafenib-induced HFSR.

## Introduction

Sorafenib is a multikinase inhibitor that targets the receptor tyrosine and serine/threonine kinases involved in tumor progression and tumor angiogenesis.^1–4^ It has been approved in many countries as frontline therapy for patients with advanced hepatocellular carcinoma, advanced renal cell carcinoma, and metastatic thyroid carcinoma. For most of these patients, sorafenib is the only treatment producing favorable therapeutic effects. However, its association with a high incidence of hand–foot skin reaction (HFSR) hampers the clinical application of this anticancer drug. Clinical trial reports from ClinicalTrial.gov and other published papers have demonstrated that the incidence percentage of sorafenib-induced HFSR ranged from 30% to as high as 76%.^5–9^ For patients with severe HFSR, dose reduction or interruption is needed, which undesirably reduces the therapeutic efficiency of sorafenib or even unlooses the progression of cancer.^10,11^

Sorafenib-induced HFSR is characterized by hyper-keratosis in palmar and plantar areas.^12–15^ Hyper-keratosis, defined as stratum corneum thickening, generally results from the abnormality in epidermal homeostasis of keratinocytes, including hyper-proliferation and hyper-differentiation.^16–19^ Since numerous studies have uncovered that most drug-induced cutaneous toxicities are the aftereffects of pathways directly affecting keratinocytes,^20–22^ keratinocyte dysfunction has thus been largely speculated as the main cause of sorafenib-induced HFSR. However, intervention strategies based on such concept are proven ineffective. For instance, controlling the presence of plantar hyper-keratosis by prophylactic removal of the hyper-keratotic areas followed by the application of a moisturizing cream has little effect on severe HFSR (grade II or III).^11^ Moreover, patients with HFSR could hardly benefit from the cushioning of callused areas by means of soft or padded shoes.^11^ Therefore, dose reduction or interruption is necessary for patients bearing high-grade HFSR, which inevitably limits the therapeutic outcomes of sorafenib. The development of effective interventions based on the underlying mechanism of sorafenib-induced HFSR becomes urgently desirable.

The growth of vascular endothelial cells leads to the formation of blood vessels that supply nutrients to skin cells. Cases of crosstalk between vascular endothelial cells and keratinocytes have been previously reported. For instance, ultraviolet radiation B (UVB) could induce vascular endothelial cells to secrete nitric oxide (NO) which promotes the DNA damage and the genetic transformation of their adjacent keratinocytes.^23^ In this study, we unravel the mechanism of sorafenib-induced HFSR by revealing the crosstalk between vascular endothelial cells and keratinocytes wherein s-HBEGF released from vascular endothelial cells stabilizes sirtuin 1 (SIRT1), an essential keratinization inducer in keratinocytes, and ultimately gives rise to HFSR. Based on such mechanistic insights, we further demonstrate HBEGF neutralization antibody and classic SIRT1 inhibitor nicotinamide could reverse sorafenib-induced HFSR, hence providing potentially promising therapeutic strategies for the treatment of this toxicity.

## Results

### Vascular endothelial cells contribute to sorafenib-induced hyper-keratosis

**Fig. 1:**
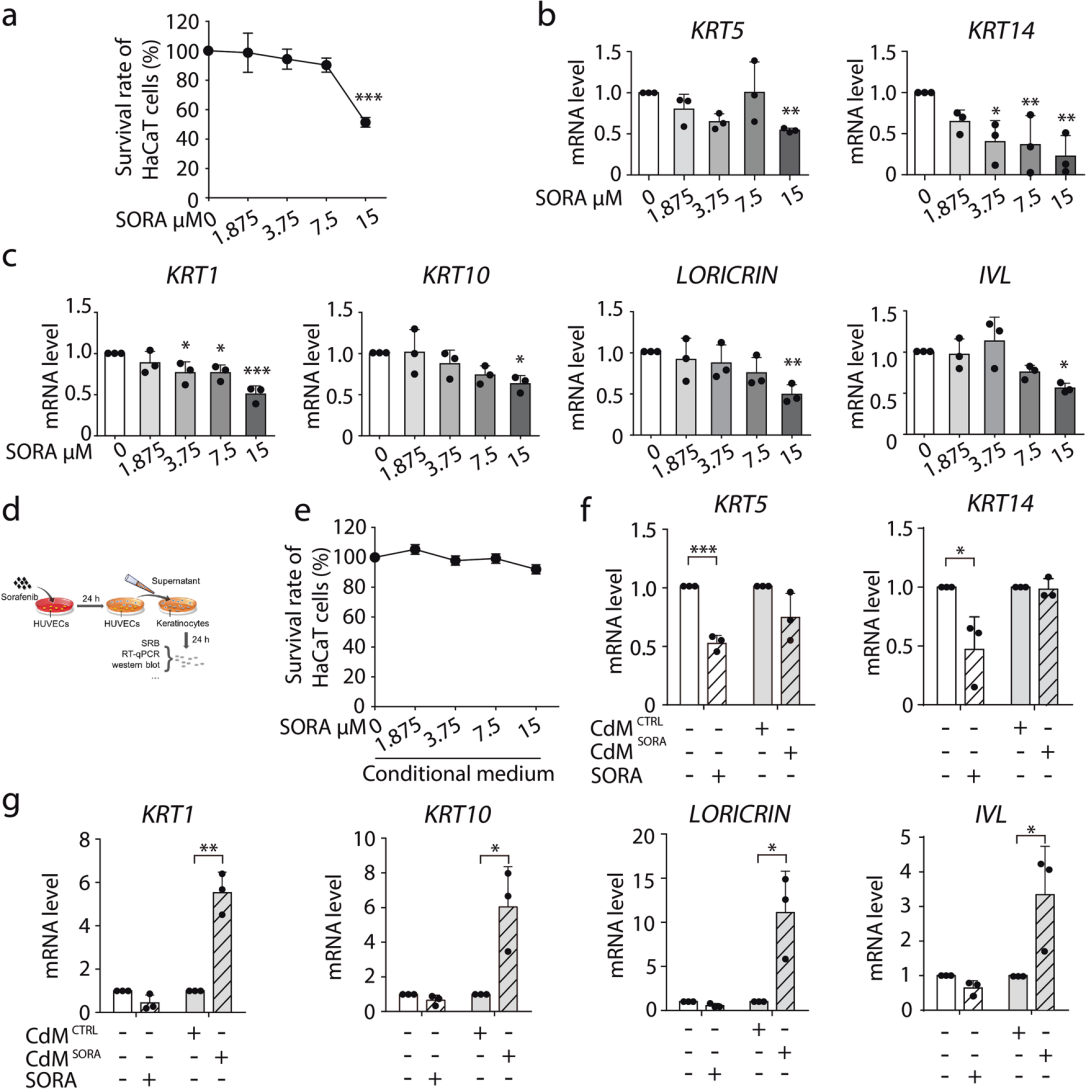
Vascular endothelial cells participate in sorafenib-induced hyper-keratosis. **a** HaCaT cells were treated with 0–15 μM sorafenib for 72 h. Cell survival rate was detected by SRB colorimetric assay (*n* = 3). **b**, **c** HaCaT cells were treated with 0–15 μM sorafenib for 24 h. The transcription levels of proliferation markers (**b**) and differentiation markers (**c**) were measured by RT-qPCR (*n* = 3). **d** The schematic diagram of conditional medium cell culture. **e** HaCaT cells were treated with supernatants from HUVECs exposed to 0–15 μM sorafenib (CdM^CTRL^ or CdM^SORA^) for 72 h. Cell survival rate was detected by SRB colorimetric assay (*n* = 3). **f**, **g** HaCaT cells were treated with supernatants from HUVECs with or without sorafenib exposure (CdM^CTRL^ or CdM^SORA^) for 24 h. **f** The transcription levels of *KRT5* and *KRT14* were measured by RT-qPCR (*n* = 3). **g** The transcription levels of *KRT1*, *KRT10*, *LORICRIN* and *IVL* were measured by RT-qPCR (*n* = 3). The results in (**a**), (**b**), (**c**), (**e**), (**f**) and (**g**) are presented as the mean ± SD. Statistical analyses were performed using unpaired two-tailed Student’s *t* test in (**f**) and (**g**). Statistical analyses were performed using one-way ANOVA with LSD post hoc test in (**a**) and when comparing the levels of *KRT14* in (**b**) and *KRT1, KRT10, LORICRIN* in (**c**) and with Dunn’s post hoc test when comparing the levels of *KRT5* in (**b**), *IVL* in (**c**). *P < 0.05; **P < 0.01; ***P < 0.001. SORA sorafenib, CdM HUVECs conditional medium, CTRL control.

Hyper-keratosis, including hyper-proliferation and hyper-differentiation, arises from the epidermal dyshomeostasis in keratinocytes.^16–19^ Given that most cutaneous toxicities induced by drugs (e.g., everolimus, adriamycin liposome, and capecitabine) are caused by cellular processes targeting keratinocytes,^20–22^ we initially sought to test the direct effect of sorafenib on keratinocytes by employing human primary keratinocytes and HaCaT cells, the immortalized human keratinocytes which largely retained the differentiation capacity of normal epidermal cells.^24^ We examined the states of proliferation and differentiation in keratinocytes, with keratin 5 (KRT5) and keratin 14 (KRT14) applied as proliferation markers, and keratin 1 (KRT1), keratin 10 (KRT10), loricrin (LORICRIN) and involucrin (IVL) characterizing differentiation.^16,25,26^ As a result, sorafenib failed to induce keratinocyte proliferation, as evidenced by the cell survival assay and proliferation marker detection (Fig. 1a, b; Supplementary information, Fig. S1a, b). Neither did sorafenib elicit keratinocyte differentiation (Fig. 1c; Supplementary information, Fig. S1c). These data implied that keratinocytes do not directly respond to sorafenib to cause hyper-keratosis.

We thus started seeking for the alternative factors that possibly drive HFSR. It is reported that in anti-angiogenesis therapies, the overall incidence of all-grade and high-grade (grade II and III) sorafenib-induced HFSR was significantly increased in patients who were additionally administered with bevacizumab, an antibody of vascular endothelial growth factor (VEGF).^27,28^ This finding accords with the known fact that vascular endothelial cells are among the cellular targets of sorafenib through the inhibition of VEGF receptors.^1^ Meanwhile, our experiments confirmed the inhibition effect of sorafenib on the proliferation of human umbilical vein endothelial cells (HUVECs) (Supplementary information, Fig. S1d). Based on these findings, we asked whether vascular endothelial cells contribute to the sorafenib-induced HFSR. To test this hypothesis, we first established a model to investigate the effect of vascular endothelial cells on keratinocytes. As illustrated in Fig. 1d, we incubated HUVECs with either dimethyl sulfoxide (DMSO) or sorafenib for 24 h, and extracted the culture media as conditional media (CdM^CTRL^ or CdM^SORA^, respectively) to treat keratinocytes for another 24 h. The effects of the media on both the keratinocyte proliferation and differentiation were subsequently examined. As a result, CdM^SORA^ did not promote the proliferation (Fig. 1e; Supplementary information, Fig. S1e) or increase proliferation markers levels (Fig. 1f; Supplementary information, Fig. S1f) of keratinocytes, indicating CdM^SORA^ did not promote the proliferation of keratinocytes. However, the mRNA levels of four differentiation markers (*KRT1*, *KRT10*, *LORICRIN* and *IVL*) significantly increased in CdM^SORA^-treated keratinocytes in comparison with those in CdM^CTRL^-treated cells (Fig. 1g; Supplementary information, Fig. S1g), suggesting that sorafenib induced keratinocyte differentiation through vascular endothelial cells.

### s-HBEGF released from vascular endothelial cells modulates sorafenib-induced hyper-keratosis

**Fig. 2:**
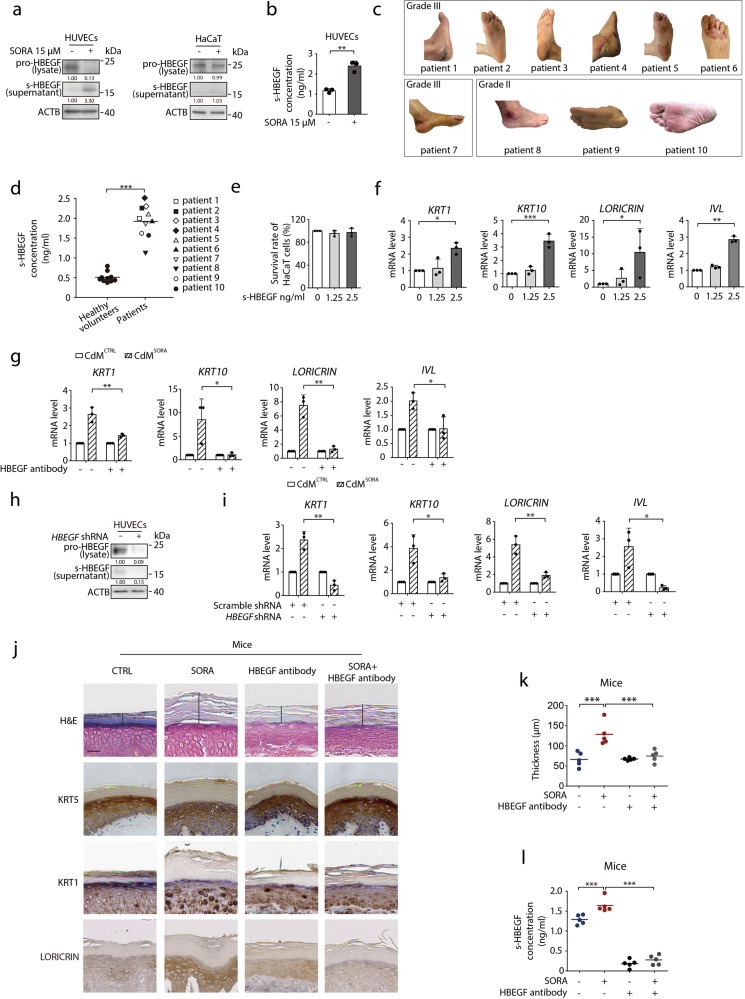
s-HBEGF governs sorafenib-induced hyper-keratosis. **a** HUVECs or HaCaT cells were exposed to 15 μM sorafenib for 24 h. The level of s-HBEGF in the supernatant or pro-HBEGF in total cell lysates was detected by western blot. **b** s-HBEGF concentrations in supernatants of HUVECs were measured by HBEGF peptide enzyme-linked immunosorbent assay (ELISA) (*n* = 3). **c** Representative photos of ten patients with HFSR of various grades. **d** s-HBEGF concentrations in the serum of ten healthy volunteers and ten patients were measured by ELISA. **e** HaCaT cells were treated with s-HBEGF for 72 h. Cell survival rate was detected by SRB assay (*n* = 3). **f** RT-qPCR analysis of *KRT1*, *KRT10*, *LORICRIN* and *IVL* in HaCaT cells treated with s-HBEGF recombinant protein for 24 h (*n* = 3). **g** HaCaT cells were treated with CdM^CTRL^ or CdM^SORA^ in the presence of HBEGF neutralization antibody for 24 h. The transcription levels of *KRT1*, *KRT10*, *LORICRIN* and *IVL* were detected by RT-qPCR (*n* = 3). **h** HUVECs were transfected with scramble shRNA or *HBEGF* shRNA via lentivirus. The level of s-HBEGF in the supernatant or pro-HBEGF in total cell lysates was detected by western blot. **i** CdM^CTRL^ or CdM^SORA^ was collected from HUVECs transfected with scramble shRNA or *HBEGF* shRNA. HaCaT cells were treated with CdM^CTRL^ or CdM^SORA^ for 24 h. The transcription levels of *KRT1*, *KRT10*, *LORICRIN* and *IVL* were detected by RT-qPCR (*n* = 3). **j**–**l** Mice were treated with HBEGF neutralization antibody (100 ng/mouse) twice a week by i.v. and/or sorafenib (100 mg/kg) daily by i.g. for 30 days (*n* = 5/group). **j** Representative H&E staining and KRT5, KRT1, LORICRIN immunohistochemistry staining were performed on the paws of mice. Scale bar, 50 μm. **k** Quantitative analysis of epidermal hyper-keratosis assessed by measuring the stratum corneum thickness (*n* = 5/group). **l** s-HBEGF concentrations in the serum of each mouse were measured by ELISA (*n* = 5/group). Densitometric values are shown as optical density after ACTB normalization using Image J. Horizontal bars in (**d**), (**k**) and (**l**) represent mean values. The results in (**b**), (**e**), (**f**), (**g**) and (**i**) are presented as the mean ± SD. Statistical analyses were performed using unpaired two-tailed Student’s *t* test in (**b**), (**d**), (**g**) and (**i**). Statistical analyses were performed using one-way ANOVA with Dunn’s post hoc test when comparing the levels of *KRT1* and *IVL* and with LSD post hoc test when comparing the levels of *KRT10* and *LORICRIN* in (**f**) and with LSD post hoc test in (**k**) and (**i**). *P < 0.05; **P < 0.01; ***P < 0.001. SORA sorafenib, CdM HUVECs conditional medium, CTRL control.

Next, we turned to identify the driving factors of keratinocyte differentiation. Mass spectrum (MS) detected several substances of interest in sorafenib-treated HUVECs medium (Supplementary information, Table S1), among which HBEGF best satisfied our criteria for further study since it (1) could be released from HUVECs, and (2) could regulate the function of keratinocytes. Previous studies revealed that HBEGF is first synthesized in a membrane-anchored form (pro-HBEGF), and then shed as a soluble structure (s-HBEGF) from ectodomain.^29–34^ To confirm the release of s-HBEGF from HUVECs, we treated the cells with sorafenib followed by western blot and enzyme-linked immunosorbent assay (ELISA) analysis. As shown in Fig. 2a, b, both assays demonstrated the increase of s-HBEGF in the supernatant of HUVECs, along with the reduction of pro-HBEGF shown from the analysis of total cell lysates (Fig. 2a left). It is worth noting that, although keratinocytes could produce s-HBEGF upon all-trans retinoic acid treatment or wound stimuli,^35,36^ we did not observe the ectodomain shedding of HBEGF in keratinocytes after sorafenib treatment (Fig. 2a right), which is in line with the fact that keratinocytes do not directly respond to sorafenib to cause hyper-differentiation.

It has been reported that enzymes matrix metalloproteinases 9 (MMP9) and matrix metalloproteinases 3 (MMP3) are involved in the cleavage of pro-HBEGF to form s-HBEGF.^37,38^ Interestingly, we also observed that sorafenib could increase both MMP9 and MMP3 protein levels in a transcription-dependent manner (Supplementary information, Fig. S2a, b). Furthermore, sorafenib-induced shedding of pro-HBEGF was blocked after treating HUVECs with marimastat, a pan-MMPs inhibitor (Supplementary information, Fig. S2c). These results suggest that MMP9 and MMP3 modulate the sorafenib-triggered release of s-HBEGF in HUVECs.

Palmoplantar keratoderma, characterized by para-keratosis and hyper-keratosis, is reportedly associated with an increased level of s-HBEGF.^39–42^ To further investigate the relationship between the s-HBEGF level and the severity of HFSR, we examined the serum from ten patients diagnosed with HFSR after sorafenib therapy. Among them, six had advanced hepatocellular carcinoma (Table 1, patients 1, 2, 3, 4, 8, and 9), two bore metastatic thyroid carcinoma (patients 6 and 7), and the rest had advanced renal cell carcinoma (patient 5 and 10). The skin biopsy of the patient 6 (grade III HFSR) was taken and indeed showed hyper-keratosis during sorafenib treatment (Supplementary information, Fig. S3). The ELISA result illustrated that patients 1–7 with grade III HFSR exhibited much higher s-HBEGF levels than patients 8–10 of grade II, while the s-HBEGF levels of the healthy volunteers appeared to be the lowest (Fig. 2c, d). These data indicated that s-HBEGF might play a key role in sorafenib-induced HFSR.

**Table 1:** Information of patients with sorafenib-induced HFSR.

No	Gender	Age	Diagnosis	HFSR grade
1	Male	73	Advanced hepatocellular carcinoma	III
2	Male	50	Advanced hepatocellular carcinoma	III
3	Male	56	Advanced hepatocellular carcinoma	III
4	Male	75	Advanced hepatocellular carcinoma	III
5	Female	75	Advanced renal cell carcinoma	III
6	Female	60	Metastatic thyroid carcinoma	III
7	Female	70	Metastatic thyroid carcinoma	III
8	Male	49	Advanced hepatocellular carcinoma	II
9	Male	48	Advanced hepatocellular carcinoma	II
10	Male	81	Advanced renal cell carcinoma	II

*HFSR* hand–foot skin reaction.

To further verify whether s-HBEGF mediates sorafenib-induced HFSR, we conducted a series of experiments in vitro. First, we treated human primary keratinocytes and HaCaT cells with s-HBEGF recombinant protein, of which the concentration was kept proportional to that in the medium of sorafenib-treated HUVECs. The proliferation was almost unchanged as evidenced by cell survival assay (Fig. 2e; Supplementary information, Fig. S4a), but a distinct increase was detected in the mRNA levels of differentiation markers, corroborating the role of s-HBEGF in facilitating keratinization (Fig. 2f; Supplementary information, Fig. S4b). Next, HBEGF neutralizing antibody was applied to block s-HBEGF from functioning. As expected, s-HBEGF neutralization reversed CdM^SORA^-promoted hyper-keratosis, as indicated by the declined mRNA levels of all four differentiation markers in HaCaT cells (Fig. 2g). In addition, we developed *HBEGF*-silenced HUVECs wherein the production of s-HBEGF was remarkably decreased (Fig. 2h). Again, the CdM^SORA^ from *HBEGF*-knockdown HUVECs failed to enhance sorafenib-induced keratinization in HaCaT cells (Fig. 2i).

Next, we developed an animal model to confirm these findings in vivo. The animal dose of sorafenib was converted in proportion from the clinical human dose.^43^ We treated the ICR mice with 100 mg/kg sorafenib daily for 30 days. Meanwhile, we established the HBEGF antibody treatment groups by administering 100 ng HBEGF neutralizing antibody per mouse to sorafenib-treated or control group twice a week, while treating the rest groups with IgG at the same dosage. After 30 days, we measured the stratum corneum thickness of each mouse. The thickness ranged from 107 to 177 µm in sorafenib-specific mice, and 53–92 µm in the sorafenib with HBEGF neutralizing antibody group (Fig. 2j, k), indicating that HBEGF neutralizing antibody almost completely reversed sorafenib-induced stratum corneum thickening, compared with the sorafenib group given IgG. We next measured the concentration of s-HBEGF in serum samples of the established mice model. As expected, the s-HBEGF level in sorafenib-treated mice was significantly higher than that in the control group, and the antibody treatment markedly reduced it (Fig. 2l). It is noteworthy that the mouse expressing the thickest corneum after sorafenib treatment possessed the highest concentration of s-HBEGF.

Finally, we sought to explore whether s-HBEGF is sufficient to induce HFSR in vivo without sorafenib treatment. The ICR mice were administered 25 ng of recombinant mouse s-HBEGF protein daily for 30 days via intravenous injection. We then analyzed the stratum corneum thickness of each mouse. The thickness ranged from 79 to 97 µm in the s-HBEGF-specific group, in comparison with that of 35–58 µm in the control group (Supplementary information, Fig. S5a, b). Moreover, the levels of differentiation markers all rose after s-HBEGF treatment (Supplementary information, Fig. S5a). These results confirmed that s-HBEGF could directly induce the keratinization in vivo.

Taken together, our findings identified the key role of s-HBEGF in mediating the crosstalk between vascular endothelial cells and keratinocytes in sorafenib-induced HFSR. More specifically, sorafenib promoted HUVECs to release s-HBEGF which reinforced the keratinization and could further cause HFSR.

### s-HBEGF governs sorafenib-induced HFSR through stabilizing SIRT1 in a JNK2-dependent manner

**Fig. 3:**
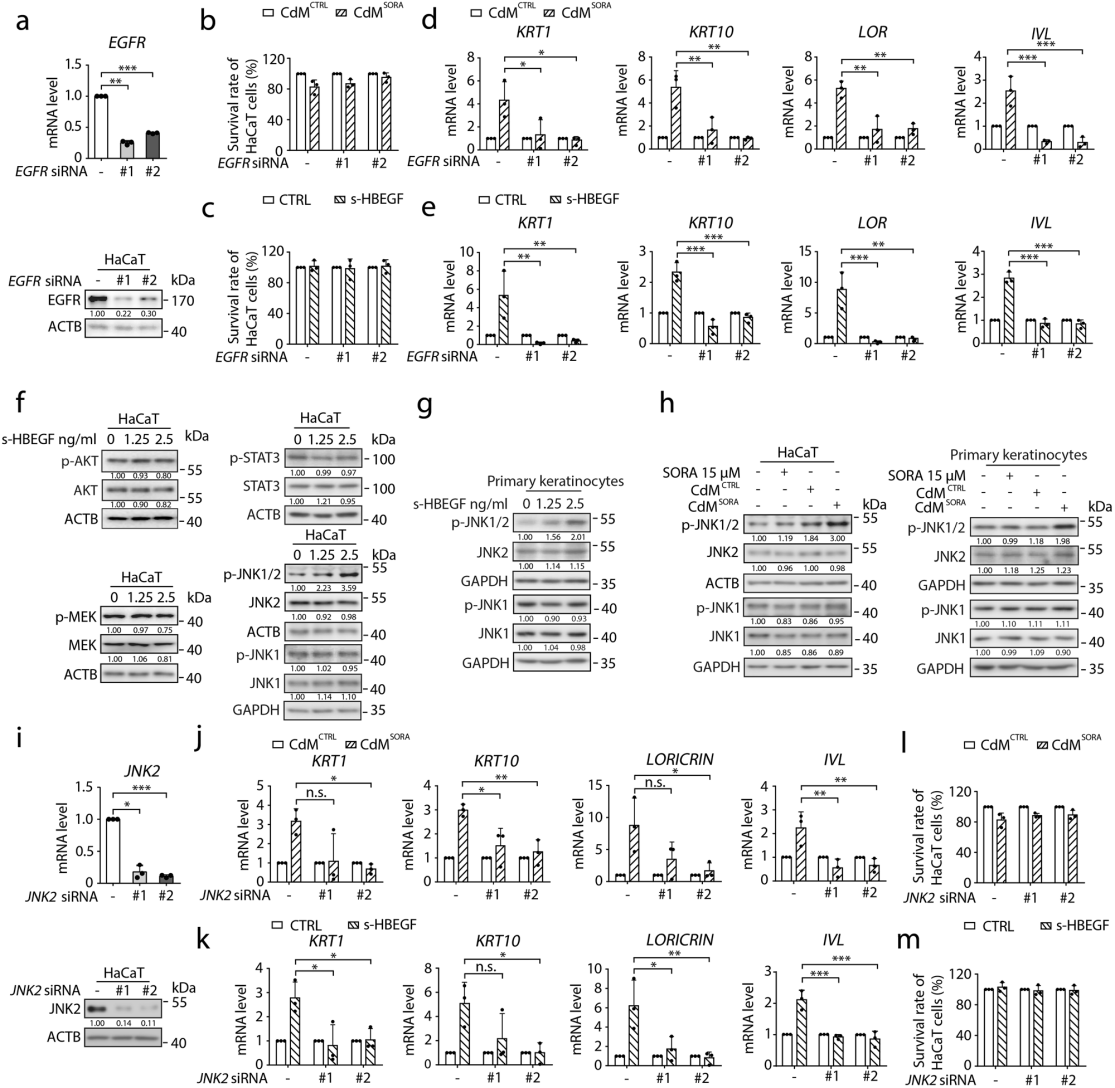
The EGFR-JNK2 axis is involved in sorafenib-induced hyper-keratosis. **a** HaCaT cells were transfected with non-targeting siRNA or siRNA targeting *EGFR*. The transcription level of *EGFR* was detected by RT-qPCR (upper panel, *n* = 3) and the expression level of EGFR was determined by western blot (lower panel). **b**–**e** HaCaT cells were transfected with non-targeting siRNA or siRNA targeting *EGFR*, followed by treatment with CdM^CTRL^ or CdM^SORA^ for 24 h (**b**, **d**) or treatment with or without s-HBEGF (2.5 ng/mL) for 24 h (**c**, **e**). The cell survival rates were measured by SRB assay (*n* = 3) (**b**, **c**). The transcription levels of *KRT1*, *KRT10*, *LORICRIN* and *IVL* were measured by RT-qPCR (*n* = 3) (**d**, **e**). **f** Relevant EGFR downstream signaling pathways were examined by western blot. **g** Human primary keratinocytes were treated with s-HBEGF and the expression levels of p-JNK1/2, JNK2, p-JNK1 and JNK1 were assessed by western blot. **h** HaCaT cells or human primary keratinocytes were treated with or without sorafenib, CdM^CTRL^ or CdM^SORA^. The expression levels of p-JNK1/2, JNK2, p-JNK1 and JNK1 were assessed by western blot. **i** HaCaT cells were transfected with non-targeting siRNA or siRNA targeting *JNK2*. *JNK2* transcription level was detected by RT-qPCR (upper panel, *n* = 3) and the expression level of JNK2 was determined by western blot (lower panel). **j–m** HaCaT cells were transfected with non-targeting siRNA or siRNA targeting *JNK2*, followed by treatment with CdM^CTRL^ or CdM^SORA^ for 24 h (**j**, **l**) or treatment with or without s-HBEGF (2.5 ng/mL) for 24 h (**k**, **m**). The transcription levels of *KRT1*, *KRT10*, *LORICRIN* and *IVL* were measured by RT-qPCR (*n* = 3) (**j**, **k**). The cell survival rates were measured by SRB assay (*n* = 3) (**l**, **m**). Densitometric values are shown as optical density after ACTB or GAPDH normalization using Image J. The results in (**a**), (**b**), (**c**), (**d**), (**e**), (**i**), (**j**), (**k**), (**l**) and (**m**) are presented as the mean ± SD. Statistical analyses were performed using one-way ANOVA with Dunn’s post hoc test in (**a**), (**i**) and when comparing the levels of *KRT1* in (**j**) and with LSD post hoc test in (**d**), (**e**), (**k**) and when comparing the levels of *KRT10*, *LORICRIN* and *IVL* in (**j**). n.s. no significance; *P < 0.05; **P < 0.01; ***P < 0.001. SORA sorafenib, CdM HUVECs conditional medium, CTRL control.

Though s-HBEGF was reported to participate in many keratinization disorders,^39–42,44^ the underlying regulating mechanism remains elusive. It is known that s-HBEGF, as a ligand, can bind to its receptor EGFR and activate downstream signal transduction.^45,46^ Inspired by this finding, we attempted to unravel the mechanism of s-HBEGF-triggered hyper-keratosis. We first generated *EGFR*-silenced HaCaT cells with two siRNAs (Fig. 3a) to examine the influence of EGFR on hyper-keratosis. As a result, silencing *EGFR* had little effect on the proliferation of keratinocytes (Fig. 3b, c) but significantly reversed the levels of keratinocyte differentiation markers elevated by CdM^SORA^ or s-HBEGF recombinant protein (Fig. 3d, e). These results suggest that EGFR functions as the s-HBEGF receptor in sorafenib-mediated hyper-keratosis.

Next, in order to identify the effector of s-HBEGF-stimulated EGFR, several classical downstream pathways, implicating AKT, STAT3, MEK, and JNK1/2, were monitored in HaCaT cells treated with s-HBEGF recombinant protein.^47–49^ Interestingly, only the level of phosphorylated JNK (p-JNK1/2) was increased (Fig. 3f, g). A similar pattern of p-JNK1/2 was observed in CdM^SORA^-treated keratinocytes as well (Fig. 3h). Since the p-JNK1 and JNK1 levels displayed no detectable change in keratinocytes (Fig. 3f–h), we turned to examine the role of JNK2 in s-HBEGF-triggered hyper-keratosis by *JNK2* silencing (Fig. 3i). As expected, the knockdown of *JNK2* attenuated the differentiation of keratinocytes under the treatment of CdM^SORA^ or s-HBEGF recombinant protein (Fig. 3j, k), without affecting the proliferation of HaCaT cells (Fig. 3l, m). Combining these findings, we concluded that activation of the EGFR-JNK2 axis may be an important link in the pathway of sorafenib-induced hyper-keratosis.

**Fig. 4:**
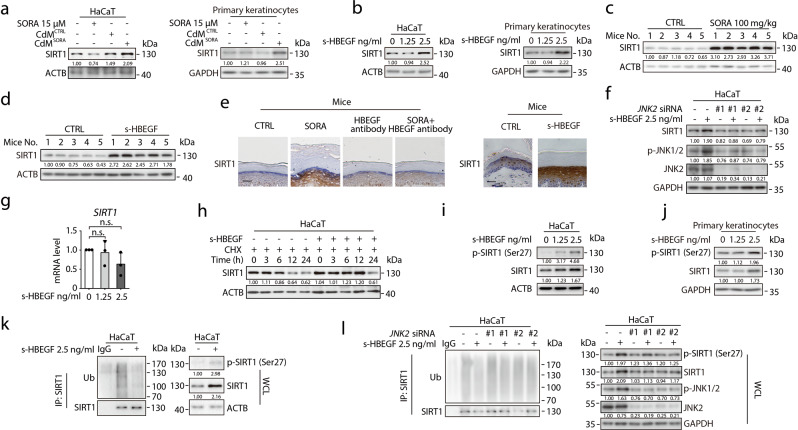
s-HBEGF stabilizes SIRT1 in keratinocytes by increasing its phosphorylation at Ser 27 via JNK2. **a** HaCaT cells or human primary keratinocytes were treated with or without sorafenib, CdM^CTRL^ or CdM^SORA^. The expression level of SIRT1 was detected by western blot. **b** HaCaT cells or human primary keratinocytes were treated with s-HBEGF for 24 h. The expression level of SIRT1 was analyzed by western blot. **c** Representative western blot indicated the expression of SIRT1 in the stratum corneum of mice in control or sorafenib-treated group (*n* = 5/group). **d** Representative western blot indicated the expression of SIRT1 in the stratum corneum of mice in control or s-HBEGF-treated group (*n* = 5/group). **e** Representative immunohistochemistry images showing SIRT1-stained paws of mice in control, sorafenib, HBEGF neutralizing antibody or combination group (left panel). Representative immunohistochemistry images showing SIRT1-stained paws of mice with or without s-HBEGF treatment (right panel). Scale bar, 50 µm. **f** HaCaT cells were transfected with non-targeting siRNA or siRNA targeting *JNK2*, followed by treatment with or without s-HBEGF (2.5 ng/mL) for 24 h. The expression levels of p-JNK1/2, JNK2 and SIRT1 were determined by western blot. **g** HaCaT cells were treated with s-HBEGF for 24 h. The transcription level of *SIRT1* was measured by RT-qPCR (*n* = 3). **h** HaCaT cells were treated with CHX with or without s-HBEGF at different time points, and SIRT1 protein level was measured by western blot. **i** HaCaT cells were treated with s-HBEGF for 24 h. The expression levels of p-SIRT1 and SIRT1 were analyzed by western blot. **j** Human primary keratinocytes were treated with s-HBEGF for 24 h. The expression levels of p-SIRT1 and SIRT1 were analyzed by western blot. **k** HaCaT cells were treated with s-HBEGF for 24 h. Cell lysates were immunoprecipitated with anti-SIRT1 antibody and probed with anti-Ub antibody or with anti-SIRT1 antibody. Protein expression levels of endogenous p-SIRT1 and SIRT1 are displayed. **l** HaCaT cells were transfected with non-targeting siRNA or siRNA targeting *JNK2*, followed by treatment with or without s-HBEGF (2.5 ng/mL) for 24 h. Cell lysates were immunoprecipitated with anti-SIRT1 antibody and probed with anti-Ub antibody or with anti-SIRT1 antibody. Protein expression levels of endogenous p-SIRT1, SIRT1, p-JNK1/2, JNK2 are displayed. Densitometric values are shown as optical density after ACTB or GAPDH normalization using Image J. The results in (**g**) are presented as the mean ± SD. Statistical analyses were performed using one-way ANOVA with LSD post hoc test in (**g**). n.s. no significance. SORA sorafenib, CHX cycloheximide, CdM HUVECs conditional medium, CTRL control, IP immunoprecipitant, WCL whole cell lysate.

Reportedly, JNK2 could phosphorylate and stabilize SIRT1,^50,51^ which might in turn stimulate peroxisome proliferator-activated receptor γ (PPARG) by coordination with CCAAT/enhancer-binding protein-α (CEBPA), direct deacetylation, or regulate the calcium-dependent pathway, and ultimately cause keratinocyte differentiation.^51,52^ Consistently, we found the expression of SIRT1 was indeed up-regulated in keratinocytes treated with either CdM^SORA^ (Fig. 4a) or s-HBEGF recombinant protein (Fig. 4b). Moreover, in the paws of sorafenib-or s-HBEGF-treated mice, we observed increased SIRT1 expression level through western blot and immunohistochemistry staining (Fig. 4c–e). In contrast, the group co-treated with HBEGF antibody and sorafenib exhibited a decreased SIRT1 level in comparison with the sorafenib treatment group (Fig. 4e).

To further corroborate that s-HBEGF-activated JNK2 could stabilize SIRT1 in keratinocytes, we first demonstrated that *JNK2* knockdown offset s-HBEGF-enhanced SIRT1 expression (Fig. 4f). Next, we examined the transcription level of *SIRT1* in HaCaT cells treated with s-HBEGF, and no significant change of *SIRT1* mRNA level was observed (Fig. 4g). A time-course experiment was subsequently performed to monitor SIRT1 degradation in the presence of protein synthesis inhibitor cycloheximide (CHX). As expected, s-HBEGF recombinant protein prolonged the half time of SIRT1 (Fig. 4h). Furthermore, we found that s-HBEGF recombinant protein could activate SIRT1 ser27 phosphorylation while inhibiting its ubiquitination (Fig. 4i–k). The stabilization of SIRT1 by s-HBEGF could be reversed by silencing *JNK2*, but not *JNK1* (Fig. 4l; Supplementary information, Fig. S6a, b). Meanwhile, we confirmed that ubiquitin-specific peptidase 22 (USP22), a classic SIRT1 deubiquitination regulator,^53,54^ was not involved in the process (Supplementary information, Fig. S7a, b).

**Fig. 5:**
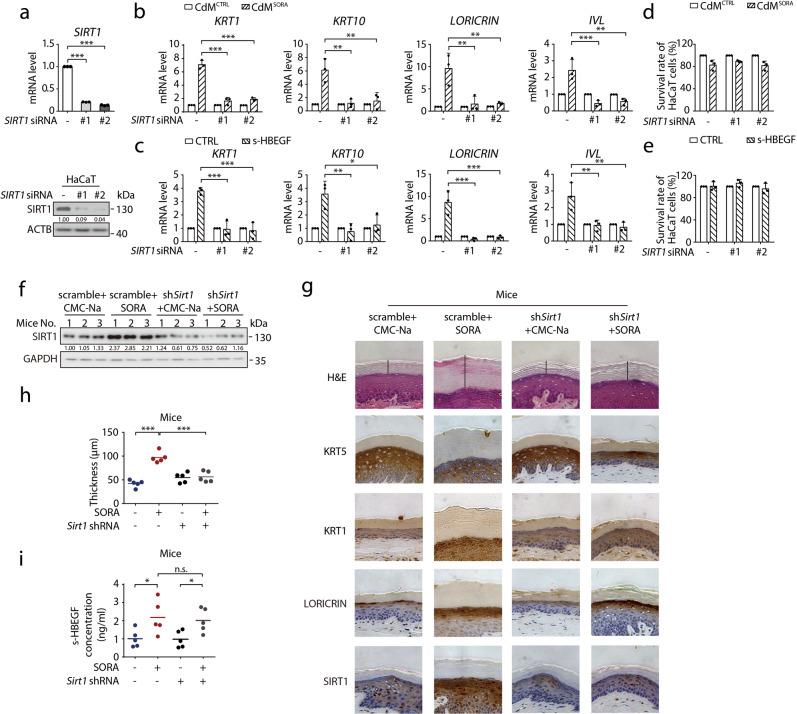
SIRT1 is involved in sorafenib-induced hyper-keratosis. **a** HaCaT cells were transfected with non-targeting siRNA or siRNA targeting *SIRT1*. *SIRT1* transcription level was detected by RT-qPCR (upper panel, *n* = 3) and the expression level of SIRT1 was determined by western blot (lower panel). **b**–**e** HaCaT cells were transfected with non-targeting siRNA or siRNA targeting *SIRT1*, followed by treatment with CdM^CTRL^ or CdM^SORA^ for 24 h (**b**, **d**) or treatment with or without s-HBEGF (2.5 ng/mL) for 24 h (**c**, **e**). The transcription levels of *KRT1*, *KRT10*, *LORICRIN* and *IVL* were measured by RT-qPCR (*n* = 3) (**b**, **c**). The cell survival rates were measured by SRB assay (*n* = 3) (**d**, **e**). **f**–**i** Mice were randomly divided into 4 groups. After injection of AAV1-sh*Sirt1* adeno virus into the paws for 2 weeks, mice were treated with CMC-Na or sorafenib (100 mg/kg) daily by i.g. for 30 days (*n* = 5/group). **f** Representative western blot indicated the expression of SIRT1 in stratum corneum of mice in each group (*n* = 3/group). **g** Representative H&E staining and KRT5, KRT1, LORICRIN, SIRT1 immunohistochemistry staining were performed on the paws of mice. Scale bar, 50 μm. **h** Quantitative analysis of epidermal hyper-keratosis assessed by measuring the stratum corneum thickness. **i** s-HBEGF concentrations in the serum of each mouse were measured by ELISA (*n* = 5/group). Densitometric values are shown as optical density after GAPDH or ACTB normalization using Image J. Horizontal bars in (**h**) and (**i**) represent mean values. The results in (**a**), (**b**), (**c**), (**d**) and (**e**) are presented as the mean ± SD. Statistical analyses were performed using one-way ANOVA with LSD post hoc test in (**b**), (**c**), (**h**) and (**i**) and with Dunn’s post hoc test in (**a**). n.s. no significance; *P < 0.05; **P < 0.01; ***P < 0.001. SORA sorafenib, CdM HUVECs conditional medium, CTRL control.

Based on these findings, we hypothesized that SIRT1, when stabilized by p-JNK2, gives rise to hyper-keratosis. To prove this, we assessed the impact of *SIRT1* silencing on sorafenib-induced hyper-keratosis (Fig. 5a). As expected, the knockdown of *SIRT1* attenuated the differentiation of keratinocytes under CdM^SORA^ or s-HBEGF recombinant protein treatment (Fig. 5b, c) without affecting the proliferation of HaCaT cells (Fig. 5d, e), suggesting that SIRT1 indeed contributed to keratinocyte differentiation in sorafenib-induced hyper-keratosis.

We next sought to determine the significance of SIRT1 in sorafenib-induced HFSR in vivo. AAV1-*Sirt1* shRNA was constructed and injected subcutaneously into the mouse paws to knock down *Sirt1* (Fig. 5f). As shown in Fig. 5g, h, the elevated stratum corneum thickness by sorafenib was reversed by the AAV1-knockdown of *Sirt1*. Moreover, histological analysis by immunohistochemistry on the thickened regions showed increased levels of SIRT1 and keratinocyte differentiation markers which were then both reduced by *Sirt1* knockdown (Fig. 5g). Furthermore, the level of s-HBEGF was increased under the treatment of sorafenib treatment and remained almost unchanged upon *Sirt1* knockdown (Fig. 5i). These findings supported that SIRT1 could mediate sorafenib-induced HFSR in vivo.

Taken together, all these results confirmed that JNK2, upon being phosphorylated by s-HBEGF-activated EGFR, could stabilize SIRT1 and give rise to hyper-keratosis.

### SIRT1 inhibitor nicotinamide mitigates sorafenib-induced HFSR in mice model

**Fig. 6:**
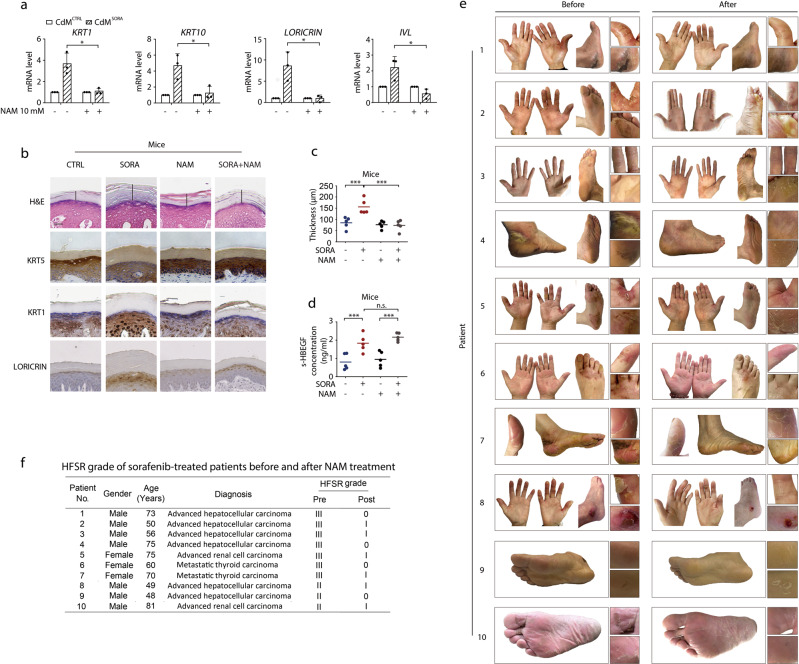
Classic SIRT1 inhibitor nicotinamide could relieve sorafenib-induced HFSR. **a** HaCaT cells were treated with or without 10 mM nicotinamide, followed by treatment with CdM^CTRL^ or CdM^SORA^ for 24 h. The transcription levels of *KRT1*, *KRT10*, *LORICRIN* and *IVL* were measured by RT-qPCR (*n* = 3). **b**–**d** Mice were treated with vehicle, sorafenib (100 mg/kg/day), nicotinamide (100 mg/kg/day) or sorafenib plus nicotinamide by intragastric administration for 30 days (*n* = 5/group). **b** Representative histopathology images show H&E-stained paws of the mice in control, sorafenib, nicotinamide or combination group. Representative immunohistochemistry images show KRT5, KRT1 or LORICRIN-stained paws of each group. Scale bar, 50 µm. **c** Thickness quantification of corneous layer of paws of the mice in control, sorafenib, nicotinamide or combination group (*n* = 5/group). **d** s-HBEGF concentrations in the serum of each mouse were measured by ELISA (*n* = 5/group). **e** Hands and feet of patients with sorafenib-induced HFSR showing hyper-keratosis before (left panel) and after (right panel) administration with 50 or 100 mg nicotinic acid (depending on HSFR grade) three times a day. **f** Diagnosis information of each patient. Horizontal bars in (**c**) and (**d**) represent mean values. The results in (**a**) are presented as the mean ± SD. Statistical analyses were performed using unpaired two-tailed Student’s *t* test in (**a**), and one-way ANOVA with LSD post hoc test in (**c**) and (**d**). n.s. no significance; *P < 0.05; ***P < 0.001. NAM nicotinamide, SORA sorafenib, CdM HUVECs conditional medium, CTRL control.

Based on the results thus far, we speculated that pharmacological inhibition of SIRT1 could be a potential treatment against sorafenib-induced HFSR. The widely used SIRT1 inhibitor nicotinamide, also referred to as vitamin B3, became our candidate of interest due to its high clinical safety and accessibility.^55–60^ We therefore combined nicotinamide with CdM^CTRL^ or CdM^SORA^ to treat HaCaT cells for 24 h, and then tested the impact of nicotinamide on sorafenib-induced cell differentiation. As shown in Fig. 6a, the increased mRNA levels of differentiation markers *KRT1*, *KRT10*, *LORICRIN* and *IVL* caused by CdM^SORA^ were almost completely reversed by nicotinamide treatment, confirming its potential therapeutic effect against sorafenib-induced HFSR.

We then set out to verify the in vivo effect of nicotinamide. Referencing its reported safe usage and treatment time in combating various diseases,^61–64^ we applied 100 mg/kg nicotinamide on tested mice with or without sorafenib treatment. As a result, nicotinamide markedly reduced both the level of differentiation markers KRT1 and LORICRIN in mouse paws and sorafenib-enhanced thickening of the stratum corneum without affecting the proliferation marker KRT5 (Fig. 6b, c). Furthermore, the elevated level of s-HBEGF by sorafenib remained almost unchanged under the co-treatment of nicotinamide (Fig. 6d), once again suggesting SIRT1 is at the downstream of s-HBEGF. Hence, we concluded that nicotinamide could be an effective intervention strategy against sorafenib-induced HFSR.

### A pilot clinical study of nicotinamide treatment in patients with sorafenib-induced HFSR

Provided the clinical safety of nicotinamide and its therapeutic effectiveness confirmed in our mice model, we proceeded to preliminarily test its clinical efficacy in treating ten cancer patients having severe HFSR (grade II or III) after sorafenib therapy. With informed consent and ethical approval in place, the patients were continuously administered with nicotinic acid, a prodrug that can transform to nicotinamide in vivo (at a dose of 50 mg for grade II HFSR or 100 mg for grade III three times daily) for 2 weeks before the examination of their HFSR state.

The results showed a distinct alleviation of hyper-keratinization and erythra in all patients (Fig. 6e) wherein grade II/III toxicities were mitigated to grade I or complete remission (Fig. 6f). In particular, male patients 1, 2, 3 and 4 with advanced hepatocellular carcinoma originally developed grade III HFSR after the treatment of sorafenib. The patients started receiving nicotinic acid in addition to their regular treatment of sorafenib. After 2 weeks, hyper-keratinization and erythra in hands and feet almost disappeared in patient 1 and were largely alleviated in patients 2 and 3. The symptoms of HFSR in feet completely remitted in patient 4. In the case of patient 5, a 75-year-old female with advanced renal cell carcinoma and grade III HFSR, the 2-week combination treatment likewise significantly reduced her symptoms of HFSR. Female patients 6 and 7 had metastatic thyroid carcinoma and grade III HFSR. The 2-week combination treatment completely healed the HFSR in patient 6 and markedly mitigated the symptoms in patient 7, especially in her feet. Male patients 8 and 9 with advanced hepatocellular carcinoma and patient 10 with advanced renal cell carcinoma all developed sorafenib-induced grade II HFSR. To our delight, palmoplantar hyper-keratinization and erythra were relieved in patients 8, 10 and completely healed in patient 9. It is noteworthy that the sorafenib dosage was not reduced during the combination treatment and nicotinamide exhibited no obvious antagonism of anticancer effect in any of the individuals. Meanwhile, the level of s-HBEGF in these patients remained almost unaffected upon nicotinamide treatment (Supplementary information, Fig. S8). Nicotinamide, therefore, could be a promising clinical treatment to combat sorafenib-induced HFSR.

## Discussion

**Fig. 7:**
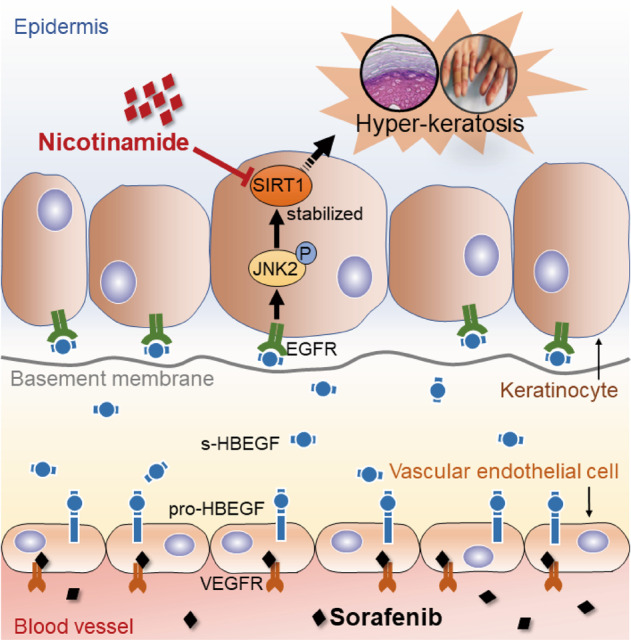
Schematic representation of the mechanism underlying sorafenib-induced hyper-keratosis. Vascular endothelial cells release s-HBEGF upon sorafenib stimulation. s-HBEGF binds to EGFR and leads to JNK2 phosphorylation in keratinocytes. The activated JNK2 subsequently stabilizes SIRT1, which eventually results in keratinization. The classic SIRT1 inhibitor nicotinamide could effectively reverse sorafenib-induced HFSR.

Our findings unprecedentedly identify the role of the vascular endothelial cell as the primary cellular target in sorafenib-induced HFSR. Mechanistically, vascular endothelial cells release s-HBEGF, a ligand that diffuses to the cell membrane of keratinocytes and binds to its receptor EGFR, which selectively stimulates the phosphorylation of downstream target JNK2. Activated JNK2 then stabilizes SIRT1, ultimately leading to hyper-keratosis, a characteristic feature of HFSR. Furthermore, SIRT1 inhibitor nicotinamide was found to be highly effective in treating sorafenib-induced HFSR (Fig. 7).

The vascular endothelial cell-keratinocyte axis is a novel insight into the mechanism underlying drug-induced cutaneous diseases, given that a majority of previous studies have only identified the keratinocyte as the primary regulator of drug-specific dermatological toxicities.^20–22^ In fact, due to the complex internal structures and functions of organisms, drugs may not always directly influence their effector cells and immediately give rise to pathological processes. Intricate cases of crosstalk were reported to occur among different types of cells at the onset of various drug toxicities, such as doxorubicin-induced cardiotoxicity and bleomycin-induced pulmonary fibrosis.^65,66^ Likewise, our results demonstrate that the primary cellular target of sorafenib-induced HFSR could be the vascular endothelial cell, rather than the commonly assumed keratinocyte. The discovery of this precise mechanism shall advance the understanding of cutaneous adverse drug reactions.

Our study also uncovers that s-HBEGF is the mediator between vascular endothelial cells and keratinocytes in sorafenib-induced HFSR. Though s-HBEGF was reported to be produced and secreted by human keratinocytes and acts as an auto/paracrine growth factor,^29,35^ we failed to detect enhanced s-HBEGF level in keratinocytes under sorafenib treatment. It may suggest that keratinocytes were not the main source of s-HBEGF production in sorafenib-induced HFSR, differing from the case of all-trans retinoic acid treatment or wound stimuli.^35,36^ In terms of functional regulation, s-HBEGF was reported to play a significant role in epidermal barrier development and maintenance.^67–69^ Studies showed that mouse mutant which expressed a high level of s-HBEGF tends to exhibit abnormally thickened skin than wild type.^70^ Furthermore, among other multikinase inhibitors that induce a high incidence of HFSR, we observed that regorafenib and anlotinib could also significantly promote the release of s-HBEGF (Supplementary information, Fig. S9), implying s-HBEGF may well participate in various multikinase inhibitor-induced HFSR and serve as a biomarker. In aggregate, our findings can not only explain the high incidence of HFSR associated with anticancer drugs, but also open new avenues for the study of cutaneous toxicities induced by similar multikinase inhibitor-based cancer treatment.

The newly discovered toxic pathway implies several potential intervention agents against sorafenib-induced HFSR, namely, the HBEGF antibody as well as the inhibitors of EGFR, JNK2, and SIRT1. Our study confirmed that HBEGF neutralizing antibody could attenuate sorafenib-induced stratum corneum thickening in mice. However, the HBEGF antibody is still under phase I trial,^71^ and the date of its clinical approval is yet undetermined. Meanwhile, the combined usage of EGFR inhibitor and sorafenib may lead to severe adverse reactions including pulmonary toxicity and hepatotoxicity,^72,73^ and no specific JNK2-inhibiting compound has yet been identified. In contrast, SIRT1 inhibitor nicotinamide, also referred to as vitamin B3, has been widely used both as a dietary supplement and a medication with an excellent safety profile.^55–60^ Reportedly, a daily dose of as high as 1500 mg was well tolerated in clinical trials for the treatment of autoimmune blistering disorders.^74^ Its effectiveness in treating sorafenib-induced HFSR was well demonstrated in our mouse models, followed by the favorable outcomes in treating ten patients clinically. Based on these positive results, we believe nicotinamide can be a promising therapeutic strategy treating sorafenib-specific HFSR adverse events. In fact, a multi-center clinical trial is currently carried out to further confirm our finding (ClinicalTrials.gov Identifier: NCT04242927).

Although the intervention treatment of HBEGF neutralization and nicotinamide effectively mitigated sorafenib-induced HFSR in mouse models and nicotinic acid even succeeded in treating ten patients, their effects on the anticancer activity of the drug are worth discussion. As a ligand of EGFR, s-HBEGF has been previously proposed as a promising target of several cancers in literature.^75–77^ Applying HBEGF neutralizing antibody or inhibiting the release of s-HBEGF may improve the clinical outcomes of patients under anticancer therapy. In our study, the combination treatment of HBEGF neutralization and sorafenib effectively reduced the toxicity, whereas its enhancement of drug efficacy warrants further study. On the other hand, we tested the influence of nicotinamide on cancer therapy in vitro. The result showed nicotinamide did not affect the sorafenib-induced death rate of the hepatocellular carcinoma cells (Supplementary information Fig. S10). In addition, after 1-month combination treatment, nicotinic acid exhibited no obvious antagonism of anticancer effect in any of the ten patients, as evidenced by their MRI result, and tumor biomarker assessment of AFP and CA199 (for advanced hepatocellular carcinoma patients) or CT assay (for patients with advanced renal cell carcinoma and metastatic thyroid carcinoma). Of note, the tumor was well controlled in eight sorafenib-sensitive patients (patients 1, 3, 4, 5, 6, 7, 9 and 10) and nicotinic acid did not promote disease progression in the rest two (patients 2 and 8). However, whether nicotinic acid influences the therapeutic effect of sorafenib over a long course merits further investigation.

In conclusion, our research reveals a new model of drug-induced cutaneous toxicity wherein sorafenib promotes keratinocyte differentiation in a vascular endothelial cell-dependent manner. We further expound the s-HBEGF/SIRT1 pathway as the distinct mechanism underlying this toxicity system. More importantly, based on the mechanistic finding, new therapeutic targets are identified for sorafenib-induced HFSR. The clinical development of nicotinamide treatment in combination with sorafenib-based cancer therapy may bring new hope to clinicians and patients, and shed new light on the study of cutaneous adverse drug reactions.

## Materials and methods

### Cell culture and reagents

Human keratinocytes (HaCaT) and human umbilical vein endothelial cells (HUVECs) were purchased from the Institute of Biochemistry and Cell Biology (Shanghai, China). They were maintained in DMEM (Gibco, 10569010) supplemented with 10% fetal bovine serum (Gibco, 10099141), 100 U/mL penicillin and 100 μg/mL streptomycin (Gibco, 10378016) in a humid atmosphere of 5% CO_2_ and 95% air at 37 °C. All cell lines were routinely tested to be negative for mycoplasma contamination. Normal human epidermal keratinocytes (NHEK) from single donor (C-12003) were purchased from Promocell (Heidelberg, Germany). They were maintained in Keratinocyte growth medium 2 (Promocell, C-20011) in a humid atmosphere of 5% CO_2_ and 95% air at 37 °C. A neutralizing monoclonal mouse anti-human HBEGF antibody (Abcam, ab89241) and normal mouse IgG (Abcam, ab188776) were used in vitro (50 ng/mL). A neutralizing monoclonal rat anti-mouse HBEGF antibody (R&D, MAB8239) and normal rat IgG (BD, 553927) were used in vivo (100 ng per mouse). Mouse HBEGF recombinant protein (Novus Biologicals, NBP2-35069-10 ug) was used in vivo (25 ng per mouse). Sorafenib (BioChemPartner, BCP01767) was prepared with DMSO (Sigma-Aldrich, D4540) in vitro and with 0.5% CMC-Na (Sigma-Aldrich, 419273) in vivo. Nicotinamide (Sigma-Aldrich, V900517) was prepared with saline in vitro and in vivo. Cycloheximide (CHX; MedChemExpress, HY-12320), regorafenib (MedChemExpress, HY-10331), Anlotinib (gift from CHIATAI TIANQING (China) Ltd) and marimastat (MedChemExpress, HY-12169) were prepared with DMSO. Recombinant human HBEGF protein (ab205523) was purchased from Abcam.

### Conditional medium

HUVECs were diluted to 1 × 10^5^ viable cells/mL, and 2 mL/well was cultured in a six-well plate. The HUVECs cultures were maintained at a 37 °C, 95% humidity and 5% CO_2_ for 24 h. Cultures were then replaced with fresh DMEM supplemented with 10% fetal bovine serum or Keratinocyte growth medium 2 and HUVECs were exposed to 15 μM sorafenib or DMSO for another 24 h. The supernatant of HUVECs was collected as conditional medium after centrifugation (1000 rpm, 5 min).

### Supernatant protein extraction

The proteins in the culture supernatants were concentrated in an Amicon Ultra-4 mL, 3 kDa Centrifugal Filter Unit (Millipore, UFC800396) by centrifugation at 4000× *g* for 60 min at 4 °C. Based on the volume of concentrated medium, the same portion of 5× loading buffer was added directly to dilute the medium to a 1× solution which was then boiled for 15 min.

### Animals

All experiments were conducted in accordance with protocols approved by the Center for Drug Safety Evaluation and Research of Zhejiang University. All mice were bred according to the protocol of the Institutional Animal Care and Use Committee (IACUC). Female ICR mice (aged 5–6 weeks and weighing 20–25 g) were purchased from Shanghai Laboratory Animal Research Center (Shanghai, China). I. Mice were randomly divided into 4 groups and treated daily via intragastric administration for 30 days: (1) control group: 0.5% CMC-Na solution; (2) sorafenib group: sorafenib at 100 mg/kg; (3) nicotinamide group: nicotinamide at 100 mg/kg; (4) sorafenib plus nicotinamide group: sorafenib at 100 mg/kg and nicotinamide at 100 mg/kg. II. Mice were randomly divided into four groups and treated daily via intragastric administration and twice a week via intravenous injection for 30 days: (1) control group: 0.5% CMC-Na solution via i.g., 0.1 mL saline solution with 100 ng normal IgG via i.v.; (2) sorafenib group: sorafenib at 100 mg/kg via i.g., 0.1 mL saline solution with 100 ng normal IgG via i.v.; (3) anti-HBEGF antibody group: 0.5% CMC-Na solution via i.g., 0.1 mL saline solution with 100 ng anti-HBEGF antibody via i.v.; (4) sorafenib plus anti-HBEGF antibody group: sorafenib at 100 mg/kg via i.g., 0.1 mL saline solution with 100 ng anti-HBEGF antibody via i.v. III. Mice were randomly divided into 2 groups and treated daily via intravenous injection for 30 days: (1) control group: 0.1 mL saline solution via i.v.; (2) s-HBEGF group: 0.1 mL saline solution with 25 ng mouse s-HBEGF via i.v. IV. *Sirt1* knockdown mouse model.

### *Sirt1* knockdown by adeno-associated virus (AAV)

An adeno-associated virus serotype 1 (AAV1), allowing for RNAi against *Sirt1* (AAV1-sh-*Sirt1*), was constructed and packaged by Vigene Biosciences (Shandong, China). The AAV1 vectors (1.29 × 10^13^ vg/mL) and AAV1-sh-*Sirt1* (1.98 × 10^13^ vg/mL) were then injected into the paws of female ICR mice (2.5 × 10^11^ vg per each paw). Two weeks after injection, the mice were treated with CMC-Na or sorafenib (100 mg/kg/day) by gavage for 30 days.

### Epidermal thickness analysis

Tissue sections with histological staining were analyzed for stratum corneum thickness. The thickness of the stratum corneum was measured using Image J software.

### Samples from healthy volunteers and patients with HFSR

The study of human serum was conducted according to the Declaration of Helsinki 2013, and approved by the institutional review board of the Hangzhou First People’s Hospital (protocol no. 2018/12-01). Blood samples (5 mL/person) were obtained from ten healthy volunteers and ten patients with HFSR at Hangzhou First People’s Hospital. Signed informed consent was obtained from all blood donors before the study.

### Human studies

The clinical efficacy of nicotinamide was tested in ten patients having severe HFSR after sorafenib therapy. Among them, six were diagnosed with advanced hepatocellular carcinoma (patient 1, 2, 3, 4, 8, 9), two with advanced renal cell carcinoma (patient 5, 10) and two with metastatic thyroid carcinoma (patient 6, 7). Baseline information of each patient was shown in Table 1. The details of grading HFSR were shown in Table 2. Patient 6 received pathology analysis. The patients were continuously administered with nicotinic acid (at a dose of 50 mg for grade II HFSR or 100 mg for grade III HFSR three times daily) and equivalent dose of sorafenib. The study was in accordance with the Declaration of Helsinki 2013, and approved by the institutional review board of the Hangzhou First People’s Hospital. Informed consent was obtained from all patients before enrollment, as well as a written agreement for photo collection of palms and soles for research purposes.

**Table 2:** NCI-CTCAE version 4.03 grading of HFSR.

Grade	Description
I	Minimal skin changes or dermatitis (e.g., erythema, edema, or hyperkeratosis) without pain
II	Skin changes (e.g., peeling, blisters, bleeding, edema, or hyperkeratosis) with pain; limiting instrumental ADL
III	Severe skin changes (e.g., peeling, blisters, bleeding, edema, or hyperkeratosis) with pain; limiting self-care ADL

*ADL* activities of daily living, *HFSR* hand–foot skin reaction, *NCI-CTCAE* National Cancer institute Common Terminology Criteria for Adverse Events.

### Plasmid constructs and transfection of shRNA

The lentiviral transfer vector pLKO.1 was purchased from Addgene plasmid (#8453). Lentivirus production was conducted by co-transfection of HEK293FT cells with three plasmids as follows: a packaging defective helper construct (pRΔ8.9; 5 μg), a construct expressing a heterologous envelope protein (pMDG-VSVG; 1 μg), and a transfer vector harboring a specific shRNA sequence (pLKO.1-shRNA; 5 μg). 3 × 10^6^ HEK293FT cells were seeded and cultured on 10 cm dish for 24 h before transfection. The cells were transfected with the plasmids using Lipofectamine 2000 (Invitrogen, 11668019) according to the manufacturer’s instructions. The medium was replaced with DMEM supplemented with 10% fetal bovine serum 24 h later and cells were cultured for another 24 h. Conditional medium was then collected and cleared of debris by filtration through a 0.45 μm filter (Millipore, France). Thereafter, 1 × 10^5^ HUVECs were seeded on each well of a 24-well plate for 24 h, and then infected by lentiviral-conditional medium with 6 μg/mL polybrene (Sigma-Aldrich, H9268). The medium was refreshed the next day and cells were cultured for another 24 h. Puromycin (1 μg/mL) (Sigma-Aldrich, P9620) was used to screen and establish the stable expression of cell lines. Transfected cells were collected for further research.

Sequence of forward oligo of sh*HBEGF* was 5′-CCGGGAGAGTCACTTTATCCTCCAACTCGAGTTGGAGGATAAAGTGACTCTCTTTTTG-3′ and sequence of forward oligo of scramble shRNA was 5′-CCGGTTCTCCGAACGTGTCACGTTTCTCGAGAAACGTGACACGTTCGGAGAATTTTTG-3′. The lentiviral packaging vectors pRΔ8.9 and pMDG-VSVG were the gifts from Dr. Lingtao Wu (University of California, USA).

### Reverse transcription quantitative PCR (RT-qPCR)

Total RNA was extracted with TRIzol Reagent according to the manufacturer’s protocol (Invitrogen, 15596026). cDNA from 1 μg RNA was prepared using a cDNA reverse transcription kit (Transgene Biotech, AT311-03). RT-qPCR was performed on a 7500 Fast System (Applied Biosystems, Singapore). The reactions were run in triplicate. The reaction mixture (20 μL) contains: cDNA obtained from reverse transcription of 1 μg RNA, 1.25 μL of forward and reverse primers (10 μM), 10 μL of iTaq™ Universal SYBR^®^ Green Supermix (Bio-Rad, 172-5125), and varied amount of DEPC water (Sangon Biotec, B501005). The samples underwent two-step amplification with an initial 3-min incubation at 95 °C, followed by incubations at 95 °C (3 s) and 60 °C (31 s) for 39 cycles. The melting curve was analyzed. Fold changes in the expression of each gene were calculated by the comparative threshold cycle (Ct) method using the formula 2^−(ΔΔCt)^. Three independent biological samples were quantified in technical duplicates and expression values were normalized to *ACTB*.

The primer sequences were as follows:

*KRT5* forward, 5′-ACAAGCAGTGTTTCCTCTGGAT-3′;

*KRT5* reverse, 5′-TAGCTTCCACTGCTACCTCCG-3′;

*KRT14* forward, 5′-CAAGATCCTGCTGGACGTGAA-3′;

*KRT14* reverse, 5′-CATGACCTTGGTGCGGATTTG-3′;

*KRT1* forward, 5′-GTCAAGTCCTCTGGTGGCAG-3′;

*KRT1* reverse, 5′-AAGGCTGGGACAAATCGACC-3′;

*KRT10* forward, 5′-CCCGGGTGTTGATCTGACTC-3′;

*KRT10* reverse, 5′-CCAGGCTTCAGCATCTTTGC-3′;

*LORICRIN* forward, 5′-TCATGATGCTACCCGAGGTTTG-3′;

*LORICRIN* reverse, 5′-CAGAACTAGATGCAGCCGGAGA-3′;

*IVL* forward, 5′-TC GCTCCTCAAGACTGTTCCTCC-3′;

*IVL* reverse, 5′-CAGGCAGTCCCTTTACAGCA-3′;

*SIRT1* forward, 5′-GACTCCAAGGCCACGGATAG-3′;

*SIRT1* reverse, 5′-GTGGAGGTATTGTTTCCGGC-3′;

*JNK1* forward, 5′-CTCGCTACTACAGAGCACCC-3′;

*JNK1* reverse, 5′-TCCCATAATGCACCCCACAG -3′;

*JNK2* forward, 5′-GCACCCTAGAAGATCCTTGAC-3′;

*JNK2* reverse, 5′-TAGCCCATACCCAGGATGAC-3′;

*EGFR* forward, 5′-AGGCACGAGTAACAAGCTCAC-3′;

*EGFR* reverse, 5′-ATGAGGACATAACCAGCCACC-3′;

*USP22* forward, 5′-CTCCTGTCTGGTCTGTGAGATG-3′;

*USP22* reverse, 5′-GAGCAACTTATACGGGATGTGA-3′;

*MMP-9* forward, 5′-CTGGAGGTTCGACGTGAAGG-3′;

*MMP-9* reverse, 5′-AGCGGTCCTGGCAGAAATAG-3′;

*MMP-3* forward, 5′-CCATCTCTTCCTTCAGGCGT-3′;

*MMP-3* reverse, 5′-ATGCCTCTTGGGTATCCAGC-3′;

*ACTB* forward, 5′-ATTCCTATGTGGGCGACGAG-3′;

and *ACTB* reverse, 5′-CCAGATTTTCTCCATGTCGTCC-3′.

### Transfection of siRNA oligonucleotides

Cells were seeded into six-well plates at 1 × 10^5^ cells per well and grown to 50%–60% confluence. Transfection was performed using Opti-MEM (Gibco, 51985034), siRNA and oligofectamine (Invitrogen, 12252011) according to the manufacturer′s recommendations. siRNA oligonucleotides were transfected at a final concentration of 12 nM. Cell culture solution was changed to complete medium for further study. The following oligonucleotides were obtained from GenePharma (Shanghai, China) as siRNAs targeting the indicated genes:

*SIRT1 #1* (5′-CAGGUCAAGGGAUGGUAUUUAUU-3′);

*SIRT1 #2* (5′-CAUGAAGUGCCUCAGAUAUUAUU-3′);

*JNK1 #1* (5′-TAAGGACTTACGTTGAAAdTdT-3′);

*JNK1 #2* (5′-CCUAAAUAUGCUGGAUAUAdTdT-3′);

*JNK2 #1* (5′-GAUUGUUUGUGCUGCAUUUdTdT-3′);

*JNK2 #2* (5′-GACUCAACCUUCACUGUCdTdT-3′);

*EGFR #1* (5′-CAAAGUGUGUAACGGAAUAdTdT-3′);

*EGFR #2* (5′-GCAAAGUGUGUAACGGAAUAGGUAU-3′);

*USP22 #1* (5′-GCUGUUUCACAAAGAAGCAUAUUCA-3′);

*USP22 #2* (5′-GCAAGGCCAAGUCCUGUAUdTdT-3′);

and Negative Control siRNA (5′-UUCUCCGAACGUGUCACGUdTdT-3′).

### Western blot

Protein lysates (30–50 μg per sample) were loaded and run on 8%, 10% or 12% SDS-polyacrylamide gels, transferred to PVDF membranes (Merck Millipore, IPVH00010) and incubated with primary antibodies overnight at 4 °C. After that, membranes were washed three times by PBS with 0.1% Tween-20 (T-PBS) for 15, 5 and 5 min, and incubated with secondary antibodies for 1 h at room temperature. After being washed three times with T-PBS, membranes were incubated with Western Lightning Plus-ECL reagent (PerkinElmer, NEL105001EA) according to the manufacturer’s protocol. Finally, membranes were exposed using Amersham Imager 600 (General Electric Company, USA). The following antibodies were used: anti-β-ACTIN (Diagbio, db10001), anti-GAPDH (Diagbio, db106), anti-HBEGF (Santa Cruz Biotechnology, sc-28908), anti-EGFR (HUABIO, ER1512-6), anti-JNK1 (Abcam, ab110724), anti-JNK2 (HUABIO, ET1610-11), anti-p-JNK1 (Thr183 & Tyr185) (Abcam, ab215208), anti-p-JNK1/2 (Thr183 & Tyr185) (Abcam, ab4821), anti-SIRT1 (HUABIO, ER130811), anti-p-SIRT1(Ser27) (Affinity, AF4484), anti-MMP9 (HUABIO, ET1704-69), anti-MMP3 (HUABIO, ET1705-98), anti-Ub (Santa Cruz Biotechnology, sc-9133), anti-p-AKT1/2/3 (Thr308) (Santa Cruz Biotechnology, sc-16646-R), anti-AKT1/2/3 (Santa Cruz Biotechnology, sc-8312), anti-STAT3 (Santa Cruz Biotechnology, sc-482), anti-p-STAT3 (Tyr705) (Santa Cruz Biotechnology, sc-7993), anti-MEK-1/2 (Santa Cruz Biotechnology, sc-436), anti-p-MEK-1/2 (Ser218/222) (Santa Cruz Biotechnology, sc-7995) and HRP-labeled secondary antibodies (MultiSciences Biotech, GAR007, GAM007, and RAG007).

### Immunoprecipitation assay

Cells were collected in lysis buffer: 50 mM Tris-HCl (pH 7.5), 150 mM NaCl, 1 mM EDTA, 1% NP-40; protease inhibitor cocktail (Cell Signaling Technology, 5871) added before use. The lysate was centrifuged and immunoprecipitated with 20 μL protein A/G Plus-agarose (Santa Cruz Biotechnology, sc-2003) and a primary antibody at 4 °C for overnight. The following primary antibodies were used: anti-SIRT1 (HUABIO, ET-1603-3, 2 µg per 500 µg of total protein). Precipitated proteins as well as initial whole-cell lysates were analyzed using western blot, as described above.

### Histological and immunohistochemical staining

The paws were harvested from mice under different treatments. For H&E staining, the specimens were fixed in formalin (F8775, Sigma-Aldrich), and embedded in paraffin before being cut into 4 μm slices. After de-waxing and rehydration, the tissue sections were stained in hematoxylin (C0105, Beyotime) for 8 min and were washed with running tap water for 5 min. They were differentiated in 1% acid alcohol for 8 s and washed with running tap water for 5 min. Next, the sections were stained in eosin (C0105, Beyotime) for 30 s. Finally, the sections were dehydrated and mounted using neutral resins to visualize the pattern in the paws. For immunohistochemical staining, the tissue sections were pretreated with 3% H_2_O_2_ (PV-6001, ZSGB-BIO) at room temperature for 10 min and blocked with 10% goat serum (Gibco, 16210064) for 15 min after dewaxing, rehydration and antigen retrieval. The SIRT1, KRT1, KRT5 or LORICRIN expression profile in mouse paws was determined by incubating the sections with a blocking solution containing anti-SIRT1 (HUABIO, ER130811) (1:200), anti-Cytokeratin 1 (Abcam, ab93652) (1:500), anti-Cytokeratin 5 (Abcam, ab75869) (1:800) or anti-Loricrin (Abcam, ab183646) (1:200) at 4 °C overnights. The primary antibody was recognized by the horseradish peroxidase (HRP)-conjugated secondary antibody (PV-6001, ZSGB-BIO) and peroxidase substrate DAB kit (ZLI-9017, ZSGB-BIO), and nuclei were stained in hematoxylin for 8 min and were washed with running tap water for 5 min. They were differentiated in 1% acid alcohol for 8 s and washed with running tap water for 5 min. The histological and immunohistochemical images were observed and captured under a light microscope (Olympus, Japan).

### ELISA assay

The contents of s-HBEGF in whole blood sample of mice or culture medium of HUVECs and whole blood sample of patients were determined with a Mouse HBEGF ELISA kit (Shanghai Jing Kang BIO, JLC3894) or a Human HBEGF ELISA kit (Shanghai Jing Kang BIO, JLC6980) according to the manufacturer’s instructions, respectively. Culture medium or whole blood sample was centrifuged at 1000× *g* for 20 min to collect the supernatant. Supernatant samples or serial dilutions of standard recombinant HBEGF were added at 50 μL/well in 96-well ELISA plate. After that, 100 μL of horseradish peroxidase (HRP)-labeled detection antibody was added to the standard well or the sample well, and the reaction wells were sealed with a sealing plate membrane before being incubated at 37 °C in a water bath or incubator for 60 min. Subsequently, the liquid in each well was discarded, and the wells were filled with 350 μL washing solution and let stand for 1 min before the washing solution being removed. The washing was repeated for five times. Then, 50 μL each of the substrates A and B were added to each well, and incubated at 37 °C for 15 min in the dark. Finally, 50 μL of the stop solution was added to each well, and the absorbance was read at a wavelength of 450 nm within 15 min. The absorbance values were converted to corresponding values in ng/mL based on the linear regression transformation of the standard curve.

### Cell survival assay

Cell survival rate was assessed using a sulforhodamine B (SRB; Sigma-Aldrich, S1402) colorimetric assay as previously described.^78^ The absorbance at 510 nm was measured using a Multiscan Spectrum (Thermo Electron Corporation Marietta) until the absorbance values remained stable. Assays were performed in triplicate via three independent experiments.

### LC-MS/MS analysis

Protein lysates were separated on 12% SDS-polyacrylamide gels. The gel pieces were cut from SDS PAGE and then sent to Shanghai Applied Protein Technology Co. Ltd for MS/MS analysis. The in-gel proteins were reduced with dithiothreitol (10 mM DTT/100 mM NH_4_HCO_3_) for 30 min at 56 °C, then alkylated with iodoacetamide (200 mM IAA/100 mM NH_4_HCO_3_) in the dark at room temperature for 30 min. Gel pieces were briefly rinsed with 100 mM NH_4_HCO_3_ and acetonitrile (ACN) each. Gel pieces were digested overnight with 12.5 ng/μL trypsin in 25 mM NH_4_HCO_3_. The peptides were extracted three times with 60% ACN/0.1% trifluoroacetic acid (TFA). The extracts were pooled and dried completely by a vacuum centrifuge. LC-MS/MS analysis was performed on a Q Exactive mass spectrometer (Thermo Scientific, USA) that was coupled to Easy nLC (Proxeon Biosystems, now Thermo Fisher Scientific, USA) for 60 min. The mass spectrometer was operated in positive ion mode. MS data were acquired using a data-dependent top10 method dynamically choosing the most abundant precursor ions from the survey scan (300–1800 m/z) for high energy collision dissociation (HCD) fragmentation. Automatic gain control (AGC) target was set to 3e6, and maximum inject time to 10 ms. Dynamic exclusion duration was 40.0 s. Survey scans were acquired at a resolution of 70,000 at m/z 200. Resolution for HCD spectra was set to 17,500 at m/z 200, and isolation width was 2 m/z. Normalized collision energy was 30 eV and the underfill ratio, which specifies the minimum percentage of the target value likely to be reached at maximum fill time, was defined as 0.1%. The instrument was run with peptide recognition mode enabled. MS/MS spectra were searched using MASCOT engine (Matrix Science, UK; version 2.2) against a nonredundant International Protein Index arabidopsis sequence database v3.85 (released at September 2011; 39679 sequences) from the European Bioinformatics Institute (http://www.ebi.ac.uk/). For protein identification, the following options were used: peptide mass tolerance = 20 ppm, MS/MS tolerance = 0.1 Da, enzyme = trypsin, missed cleavage = 2, fixed modification: carbamidomethyl (C), variable modification: oxidation (M).

### Statistical analyses

Statistical analysis was performed using SPSS 21.0 software. All data were expressed as the mean value or mean value ± standard deviation (SD). When comparing two groups, Student’s *t* test (unpaired, two-tailed) was performed. For experiments in which one variable was analyzed for multiple conditions, one-way ANOVA was performed. If the differences among groups were significant (*P* < 0.05), Dunn’s test was used for post hoc comparisons between pairs of groups. Otherwise, LSD post hoc test was used.

## Supplementary information

Supplementary Figure S1

Supplementary Figure S2

Supplementary Figure S3

Supplementary Figure S4

Supplementary Figure S5

Supplementary Figure S6

Supplementary Figure S7

Supplementary Figure S8

Supplementary Figure S9

Supplementary Figure S10

Supplementary Table S1
