# Supplementary material for: s-HBEGF/SIRT1 circuit-dictated crosstalk between vascular endothelial cells and keratinocytes mediates sorafenib-induced hand–foot skin reaction that can be reversed by nicotinamide
Source: Cell Res. 2020 Apr 15;30(9):779–93. doi: 10.1038/s41422-020-0309-6 (PMC7608389; doi:10.1038/s41422-020-0309-6)
Supplement: Supplementary file 1 — Supplementary Figure S1 [file 41422_2020_309_MOESM1_ESM.pdf]

Supplementary Figure S1

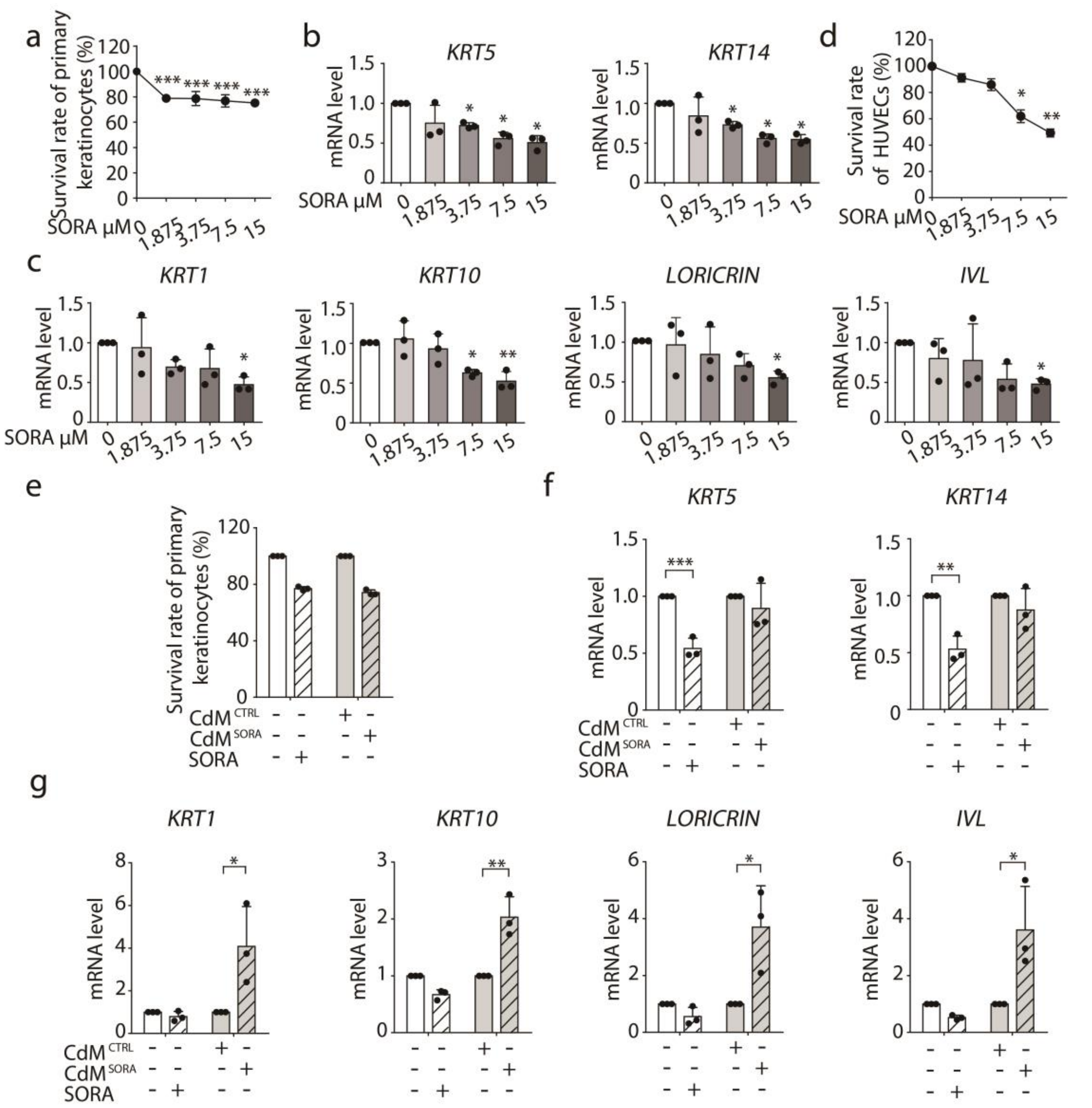

Fig. S1 Vascular endothelial cells participate in sorafenib-induced hyper-keratosis in human primary keratinocytes.

a Human primary keratinocytes were treated with 0-15 μM sorafenib for 24 h. Cell survival rate was detected by SRB colorimetric assay (N = 3). **b**, **c** Human primary keratinocytes were treated with 0-15 μM sorafenib for 24 h. The transcription levels of proliferation markers (**b**) and differentiation markers (**c**) were measured by RT-qPCR (N = 3). **d** HUVECs were treated with 0-15 μM sorafenib for 72 h. Cell survival rate was detected by SRB colorimetric assay (N = 3). **e** Human primary keratinocytes were treated with supernatants from HUVECs exposed to 0 or 15 μM sorafenib (CdM^{CTRL} or CdM^{SORA}) for 24 h. Cell survival rate was

detected by SRB colorimetric assay (N = 3). **f**, **g** Human primary keratinocytes were treated with supernatants from HUVECs with or without sorafenib exposure (CdM^{CTRL} or CdM^{SORA}) for 24 h. (**f**) The transcription levels of *KRT5* and *KRT14* were measured by RT-qPCR (N = 3). (**g**) The transcription levels of *KRT1, KRT10, LORICRIN* and *IVL* were measured by RT-qPCR (N = 3). The results in (**a**), (**b**), (**c**), (**d**), (**e**), (**f**) and (**g**) are presented as the mean ± SD. Statistical analyses were performed using unpaired two-tailed Student's t-test in (**f**) and (**g**). Statistical analyses were performed using one-way ANOVA with LSD post hoc test in (**a**) and when comparing the levels of *KRT10* in (**c**) and with Dunn's post hoc test in (**b**), (**d**) and when comparing the levels of *KRT1, LORICRIN* and *IVL* in (**c**). **P* < 0.05; ***P* < 0.01; *** *P* < 0.001. SORA, sorafenib; CdM, HUVECs conditional medium; CTRL, control; SRB, sulforhodamine B.
