# Supplementary material for: s-HBEGF/SIRT1 circuit-dictated crosstalk between vascular endothelial cells and keratinocytes mediates sorafenib-induced hand–foot skin reaction that can be reversed by nicotinamide
Source: Cell Res. 2020 Apr 15;30(9):779–93. doi: 10.1038/s41422-020-0309-6 (PMC7608389; doi:10.1038/s41422-020-0309-6)
Supplement: Supplementary file 2 — Supplementary Figure S2 [file 41422_2020_309_MOESM2_ESM.pdf]

Supplementary Figure S2

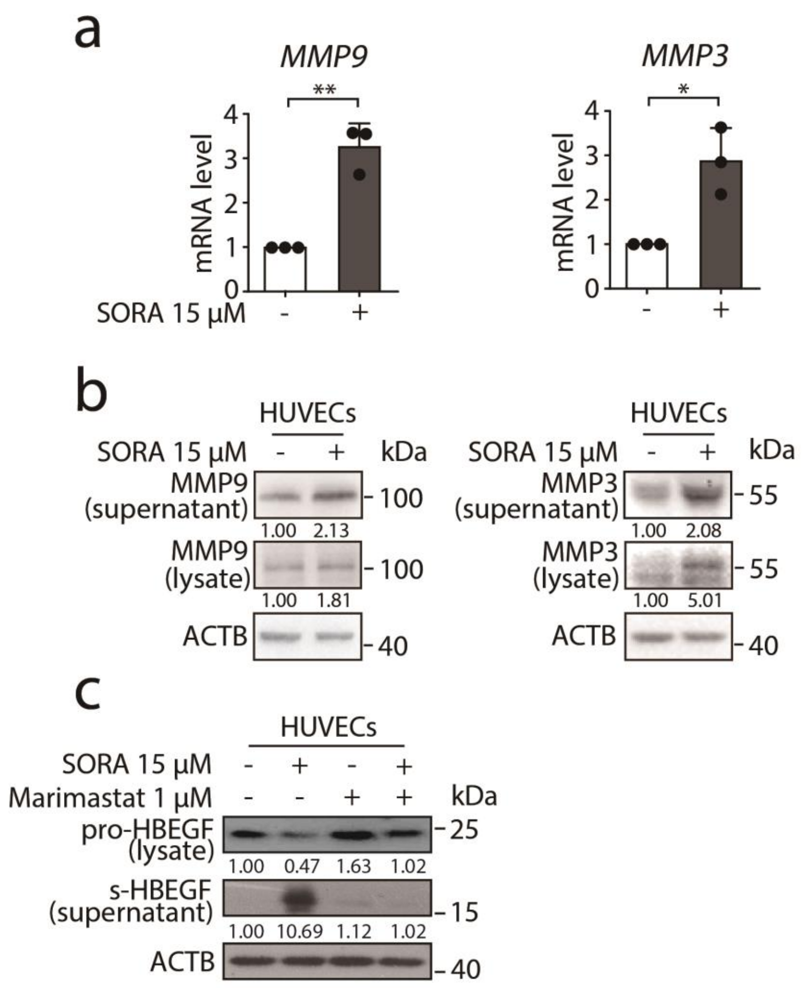

Fig. S2 Sorafenib increases protein levels of MMP9 and MMP3 in a transcription-dependent manner.

a HUVECs were treated with 15 μM sorafenib for 24 h. *MMP9* and *MMP3* transcription levels were detected by RT-qPCR (N = 3). The result is presented as the mean ± SD. Statistical analysis was performed using unpaired two-tailed Student's t-tests. *$P < 0.05$; **$P < 0.01$. **b** HUVECs were exposed to 15 μM sorafenib for 24 h. The levels of MMP9 and MMP3 in the supernatant or in total cell lysates were detected by western blot. **c** HUVECs were treated with or without sorafenib or marimastat for 24 h. The level of s-HBEGF in the supernatant or pro-HBEGF in total cell lysates was detected by western blot. Densitometric values are shown as optical density after ACTB normalization using Image J. SORA, sorafenib.
