# Supplementary material for: s-HBEGF/SIRT1 circuit-dictated crosstalk between vascular endothelial cells and keratinocytes mediates sorafenib-induced hand–foot skin reaction that can be reversed by nicotinamide
Source: Cell Res. 2020 Apr 15;30(9):779–93. doi: 10.1038/s41422-020-0309-6 (PMC7608389; doi:10.1038/s41422-020-0309-6)
Supplement: Supplementary file 3 — Supplementary Figure S3 [file 41422_2020_309_MOESM3_ESM.pdf]

Supplementary Figure S3

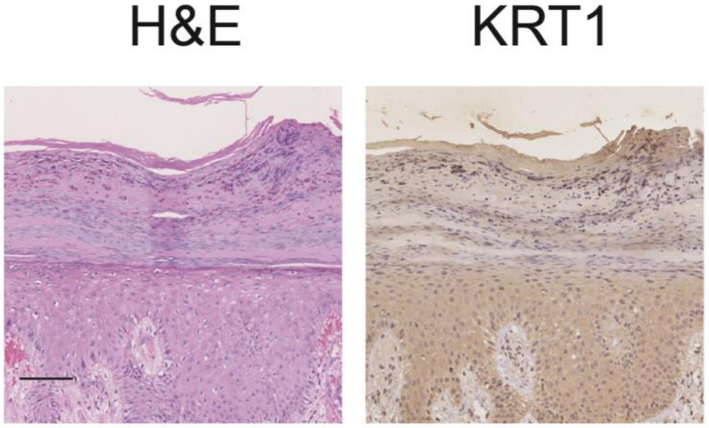

Fig. S3 Histopathologic aspect with patient skin biopsy specimens.

Skin sections from patient 6, a 60-year-old woman with metastatic thyroid carcinoma. Representative H&E staining and KRT1 immunohistochemistry staining were performed. Scale bar, 100 μm.
