# Supplementary material for: s-HBEGF/SIRT1 circuit-dictated crosstalk between vascular endothelial cells and keratinocytes mediates sorafenib-induced hand–foot skin reaction that can be reversed by nicotinamide
Source: Cell Res. 2020 Apr 15;30(9):779–93. doi: 10.1038/s41422-020-0309-6 (PMC7608389; doi:10.1038/s41422-020-0309-6)
Supplement: Supplementary file 4 — Supplementary Figure S4 [file 41422_2020_309_MOESM4_ESM.pdf]

Supplementary Figure S4

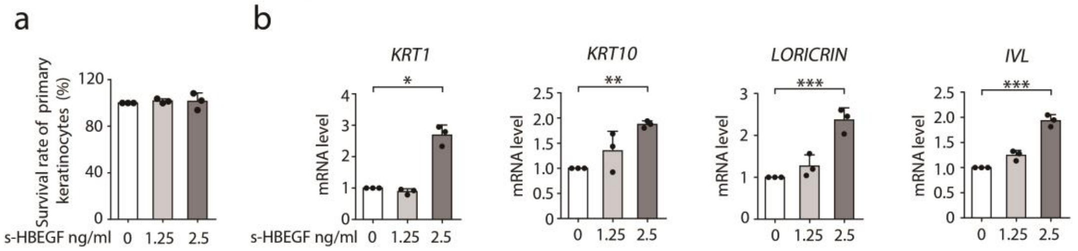

Fig. S4 s-HBEGF induces keratinization in human primary keratinocytes.

a Human primary keratinocytes were treated with s-HBEGF for 24 h. Cell survival rate was detected by SRB colorimetric assay (N = 3). **b** RT-qPCR analysis was used to measure the levels of *KRT1*, *KRT10*, *LORICRIN* and *IVL* in human primary keratinocytes treated with s-HBEGF recombinant protein for 24 h (N = 3). The results in (**a**) and (**b**) are presented as the mean ± SD. Statistical analyses were performed using one-way ANOVA with Dunn's post hoc test when comparing the levels of *KRT1* and *KRT10* and with LSD post hoc test when comparing the levels of and *LORICRIN* and *IVL* in (**b**). *$P < 0.05$; **$P < 0.01$; ***$P < 0.001$.
