# Supplementary material for: s-HBEGF/SIRT1 circuit-dictated crosstalk between vascular endothelial cells and keratinocytes mediates sorafenib-induced hand–foot skin reaction that can be reversed by nicotinamide
Source: Cell Res. 2020 Apr 15;30(9):779–93. doi: 10.1038/s41422-020-0309-6 (PMC7608389; doi:10.1038/s41422-020-0309-6)
Supplement: Supplementary file 5 — Supplementary Figure S5 [file 41422_2020_309_MOESM5_ESM.pdf]

Supplementary Figure S5

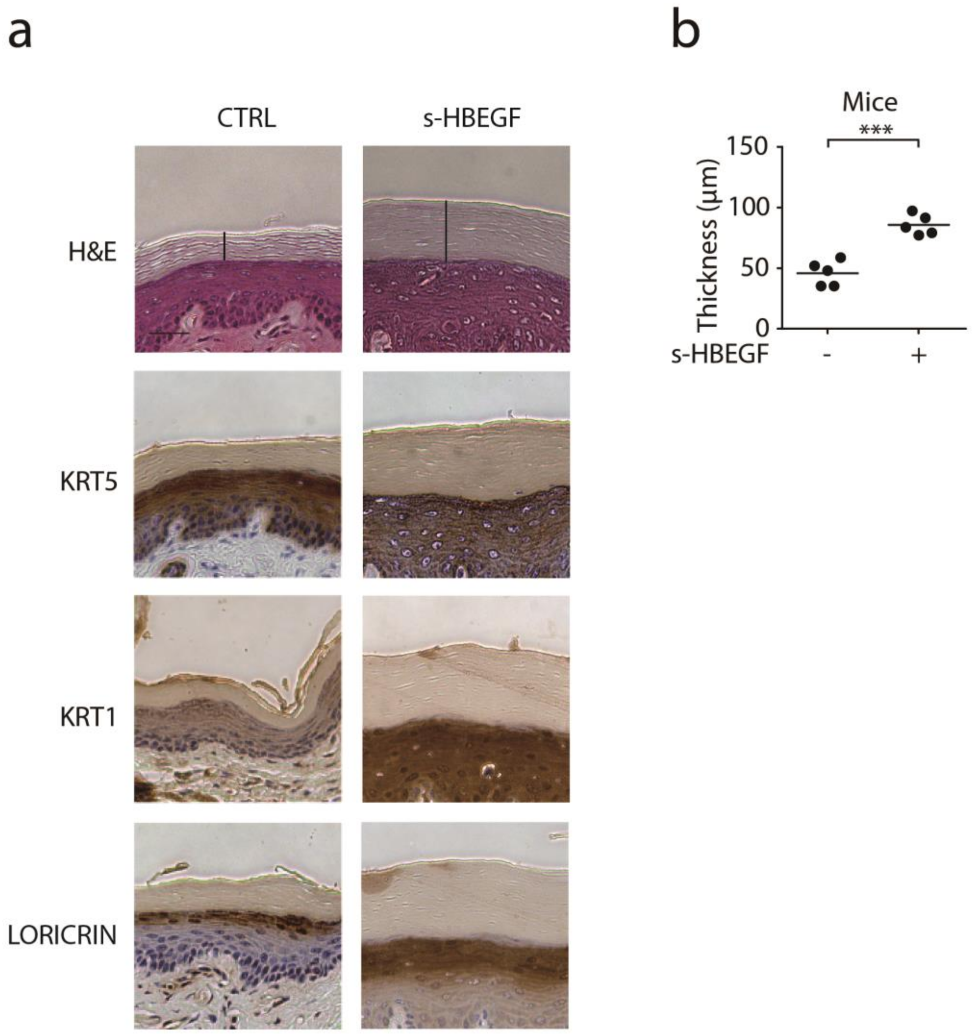

Fig. S5 s-HBEGF could induce hyper-keratinization in mice.

Mice were injected with or without s-HBEGF (25 ng per mouse) via tail vein for 30 days. **a** Representative H&E staining and KRT5, KRT1, LORICRIN immunohistochemistry staining were performed on the stratum corneum of mice. Scale bar, 50 μm. **b** Quantitative analysis of epidermal hyper-keratosis assessed by measuring the stratum corneum thickness. Horizontal bars in (b) represent mean values. Statistical analyses were performed using unpaired two-tailed Student's t-test in (**b**). ***$P < 0.001$.
