# Supplementary material for: s-HBEGF/SIRT1 circuit-dictated crosstalk between vascular endothelial cells and keratinocytes mediates sorafenib-induced hand–foot skin reaction that can be reversed by nicotinamide
Source: Cell Res. 2020 Apr 15;30(9):779–93. doi: 10.1038/s41422-020-0309-6 (PMC7608389; doi:10.1038/s41422-020-0309-6)
Supplement: Supplementary file 6 — Supplementary Figure S6 [file 41422_2020_309_MOESM6_ESM.pdf]

Supplementary Figure S6

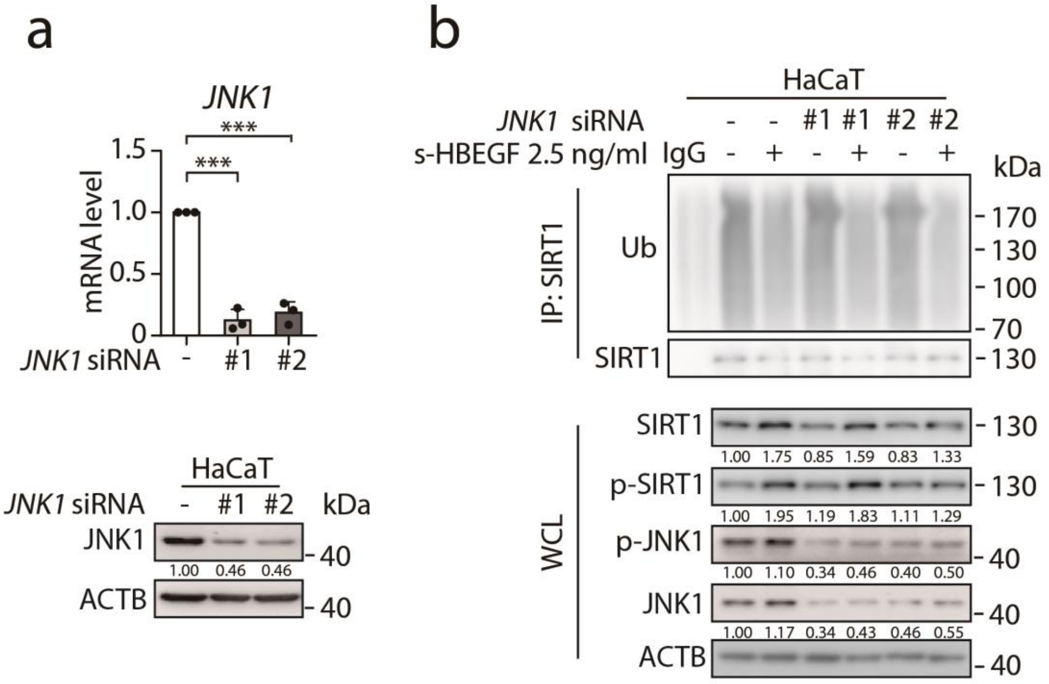

Fig. S6 s-HBEGF stabilizes keratinocyte SIRT1 independent of JNK1.

a, b HaCaT cells were transfected with non-targeting siRNA or siRNA targeting *JNK1*. (**a**) *JNK1* transcription level was detected by RT-qPCR (upper panel, N = 3) and the expression level of JNK1 was determined by western blot (lower panel). (**b**) Cell lysates were immunoprecipitated with anti-SIRT1 antibody and probed with anti-Ub antibody or with anti-SIRT1 antibody. Protein expression levels of endogenous p-SIRT1, SIRT1, p-JNK1, JNK1 are displayed. Statistical analyses were performed using one-way ANOVA with LSD post hoc test in (**a**). ****P* < 0.001. Densitometric values are shown as optical density after ACTB normalization using Image J.
