# Supplementary material for: s-HBEGF/SIRT1 circuit-dictated crosstalk between vascular endothelial cells and keratinocytes mediates sorafenib-induced hand–foot skin reaction that can be reversed by nicotinamide
Source: Cell Res. 2020 Apr 15;30(9):779–93. doi: 10.1038/s41422-020-0309-6 (PMC7608389; doi:10.1038/s41422-020-0309-6)
Supplement: Supplementary file 7 — Supplementary Figure S7 [file 41422_2020_309_MOESM7_ESM.pdf]

Supplementary Figure S7

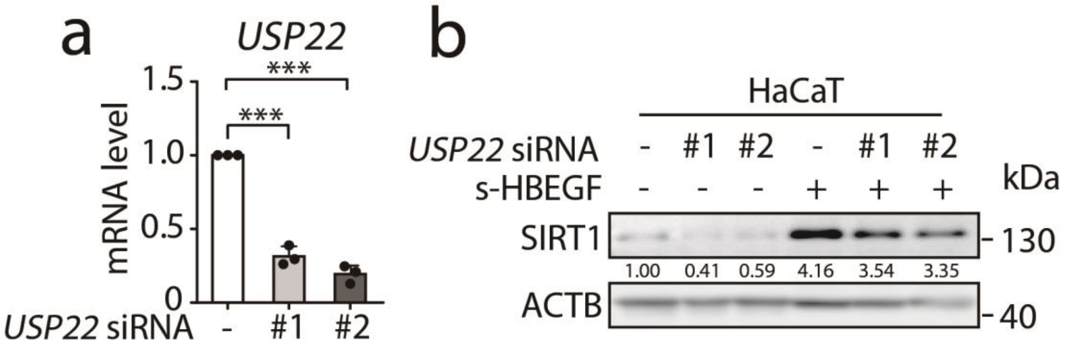

Fig. S7 s-HBEGF stabilizes keratinocyte SIRT1 independent of USP22.

a HaCaT cells were transfected with non-targeting siRNA or siRNA targeting *USP22*. *USP22* transcription level was detected by RT-qPCR (N = 3). The result is presented as the mean ± SD. Statistical analysis was performed using one-way ANOVA with LSD post hoc test. ***$P < 0.001$. **b** HaCaT cells were transfected with non-targeting siRNA or siRNA targeting *USP22*, followed by treatment with or without s-HB-EGF (2.5 ng/ml) for 24 h. The expression levels of SIRT1 were determined by western blot. Densitometric values are shown as optical density after ACTB normalization using Image J.
