# Supplementary material for: s-HBEGF/SIRT1 circuit-dictated crosstalk between vascular endothelial cells and keratinocytes mediates sorafenib-induced hand–foot skin reaction that can be reversed by nicotinamide
Source: Cell Res. 2020 Apr 15;30(9):779–93. doi: 10.1038/s41422-020-0309-6 (PMC7608389; doi:10.1038/s41422-020-0309-6)
Supplement: Supplementary file 8 — Supplementary Figure S8 [file 41422_2020_309_MOESM8_ESM.pdf]

Supplementary Figure S8

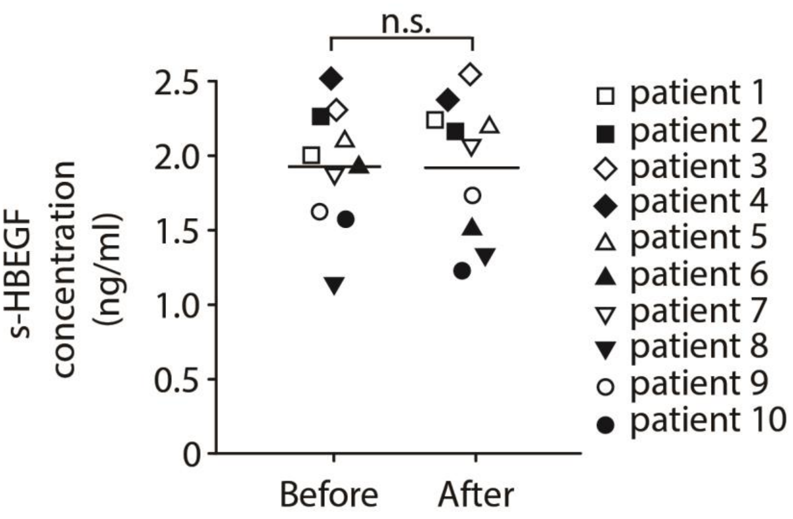

Fig. S8 The level of s-HBEGF in patients with HFSR before and after NAM treatment.

s-HBEGF concentrations of 10 patients were measured by ELISA before and after NAM treatment. ELISA, enzyme-linked immunosorbent assay; NAM, Nicotinamide. Statistical analyses were performed using unpaired two-tailed Student's t-test. n.s. = no significance.
