# Supplementary material for: s-HBEGF/SIRT1 circuit-dictated crosstalk between vascular endothelial cells and keratinocytes mediates sorafenib-induced hand–foot skin reaction that can be reversed by nicotinamide
Source: Cell Res. 2020 Apr 15;30(9):779–93. doi: 10.1038/s41422-020-0309-6 (PMC7608389; doi:10.1038/s41422-020-0309-6)
Supplement: Supplementary file 9 — Supplementary Figure S9 [file 41422_2020_309_MOESM9_ESM.pdf]

Supplementary Figure S9

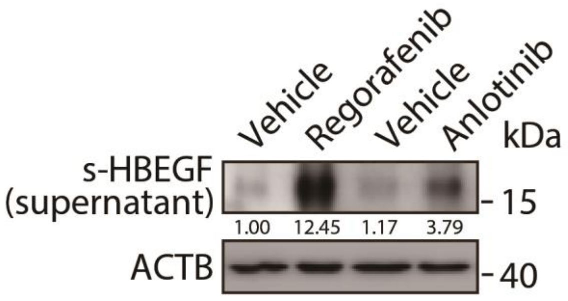

Fig. S9 The effect of regorafenib and anlotinib on the release of s-HBEGF in HUVECs. HUVECs were exposed to 8 μM regorafenib or 25 nM anlotinib for 24 h. The level of s-HBEGF in the supernatant was detected by western blot. Densitometric values are shown as optical density after ACTB normalization using Image J.
