# Supplementary material for: s-HBEGF/SIRT1 circuit-dictated crosstalk between vascular endothelial cells and keratinocytes mediates sorafenib-induced hand–foot skin reaction that can be reversed by nicotinamide
Source: Cell Res. 2020 Apr 15;30(9):779–93. doi: 10.1038/s41422-020-0309-6 (PMC7608389; doi:10.1038/s41422-020-0309-6)
Supplement: Supplementary file 10 — Supplementary Figure S10 [file 41422_2020_309_MOESM10_ESM.pdf]

Supplementary Figure S10

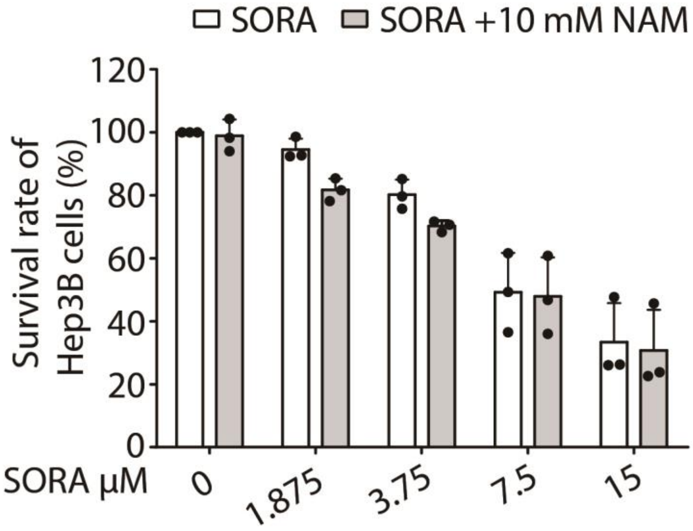

Fig. S10 Nicotinamide does not affect sorafenib-induced Hep3B cell death.

Hep3B cells were treated with 0-15 μM sorafenib with or without nicotinamide 10 μM for 72 h. Cell survival rate was detected by SRB colorimetric assay (N = 3). Data are expressed as the mean ± SD. SORA, sorafenib; SRB, sulforhodamine B, NAM, nicotinamide.
