# Supplementary material for: s-HBEGF/SIRT1 circuit-dictated crosstalk between vascular endothelial cells and keratinocytes mediates sorafenib-induced hand–foot skin reaction that can be reversed by nicotinamide
Source: Cell Res. 2020 Apr 15;30(9):779–93. doi: 10.1038/s41422-020-0309-6 (PMC7608389; doi:10.1038/s41422-020-0309-6)
Supplement: Supplementary file 11 — Supplementary Table S1 [file 41422_2020_309_MOESM11_ESM.pdf]

Table. S1. The number of unique and total peptides for the supernatant of CdM^{CRTL} and CdM^{SORA} via LC-MS/MS analysis

Accession	Description	# unique peptides	# total peptides	# unique Peptides + sorafenib	# total Peptides + sorafenib
P07355	ANXA2	1	1	15	15
P00558	PGK1	0	0	9	9
P09211	GSTP1	2	2	9	9
P04083	ANXA1	1	1	8	8
P28482	MAPK1	0	0	8	10
P09429	HMGB1	1	1	4	5
Q01469	FABP5	0	0	4	4
P0DP23	CALM1	0	0	4	4
P07339	CTSD	0	0	4	4
P08758	ANXA5	1	1	4	5
Q99075	HBEGF	0	0	4	4
Q9UQ80	PA2G4	0	0	3	3
Q9Y376	CAB39	0	0	3	3
Q16270	IGFBP7	10	10	2	2
Q9Y5Z4	HEBP2	0	0	2	2
P18065	IGFBP2	0	0	1	1
Q04756	HGFAC	0	0	1	1

Abbreviation: CRTL, control; SORA, sorafenib; CdM, HUVECs conditional medium.
